# Shared donor–recipient γδ T‐cell phenotypic and repertoire features associate with cytomegalovirus reactivation after allogeneic haematopoietic stem cell transplantation

**DOI:** 10.1002/cti2.70068

**Published:** 2025-12-17

**Authors:** Paula Hahn, Arwen Stikvoort, Faisal Alagrafi, Ahmed Gaballa, Martin Solders, Sofie Vonlanthen, Johan Karlsson Törlén, Lucas C M Arruda, Thomas Poiret, Michael Uhlin

**Affiliations:** ^1^ Centre for Hematology and Regenerative Medicine, Department of Medicine, Huddinge Karolinska Institutet Huddinge Sweden; ^2^ Healthy Aging Research Institute King Abdulaziz City for Science and Technology Riyadh Kingdom of Saudi Arabia; ^3^ Department of Clinical Immunology and Transfusion Medicine Karolinska University Hospital Huddinge Sweden; ^4^ Cell Therapy and Allogeneic Stem Cell Transplantation Karolinska University Hospital Huddinge Sweden; ^5^ Department of Laboratory Medicine Karolinska Institutet Huddinge Sweden

**Keywords:** γδ T‐cells, ATG, CMV, HSCT

## Abstract

Allogeneic haematopoietic stem cell transplantation (HSCT) is the only curative therapy for patients diagnosed with acute myeloid leukaemia (AML) and myelodysplastic syndrome (MDS). γδ+ T‐cells, a rare subpopulation of T‐cells with both innate and adaptive anticancer functions, have been understudied and the role of different γδ+ T‐cell subsets after HSCT is unclear.

**Objectives:**

We aimed to characterise the γδ+ T‐cells reconstitution post‐HSCT, investigating their association with graft‐versus‐host disease (GvHD) treatment and occurrence, and cytomegalovirus (CMV) reactivation.

**Methods:**

Peripheral blood samples from AML and MDS patients and their respective donors were immunophenotyped by flow cytometry before and at several time points after HSCT. Additionally, next‐generation sequencing of the *TRG* locus was performed to assess clonal dynamics and repertoire diversity.

**Results:**

Early after HSCT, γδ+ T‐cells showed a similar phenotype compared to pre‐HSCT, and stable repertoires were observed up to 6 months post‐HSCT. Patients with acute GVHD presented a higher frequency of CD8 in γδ+ T‐cells, and γδ+ T‐cell and subsets frequencies were not affected by anti‐thymocyte globulin prophylaxis. Donor‐derived Vδ2+ T‐cell central memory phenotype was associated with a reduced risk of CMV reactivation in the recipient and with repertoire disturbance. Effector memory cells displaying a specific HLA‐DR+ CD86+ phenotype were associated with CMV reactivation and a higher proportion of hyperexpanded clonotypes prior to transplantation.

**Conclusion:**

Overall, Vδ1+ and Vδ2+ T‐cell phenotypic profiles and γδ TCR repertoire remain stable post‐HSCT. This stability is disrupted by CMV reactivation, which is potentially associated with γδ+ T‐cells of donor origin and γδ TCR repertoire.

## Graphical abstract

HSCT does not affect key phenotypic profiles of Vδ1+ and Vδ2+ subsets of γδ T‐cells, unless CMV reactivation occurs in which case repertoire disturbance and higher effector memory phenotype of donor‐derived γδ T‐cells are observed. Moreover, ATG does not affect the viability of γδ T‐cells post‐HSCT.

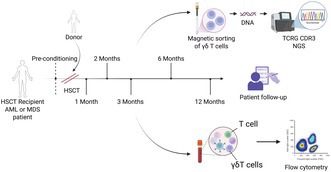

## Introduction

Haematopoietic stem cell transplantation (HSCT) is the most effective and sometimes the only curative treatment for certain hematologic malignancies, most commonly acute myeloid leukaemia (AML).^1^ However, relapse remains one of the major challenges following HSCT, occurring in up to 50% of cases.^1^ Additional complications include graft‐versus‐host disease (GvHD) and viral reactivations.^2^, ^3^ These events are directly related to the immune environment post‐HSCT, highlighting the critical importance of an efficient immune reconstitution for patient survival and long‐term well‐being.^2^, ^4^, ^5^

Studying the timely recovery of T‐cells is essential to understanding and addressing these complications, since delays or impairments in this process are closely linked to increased morbidity and mortality.^6^ Deciphering the recovery of a diverse and functional T‐cell receptor (TCR) repertoire and integrating it with the occurrence of clinical adverse events will allow better patient management in the future by optimising conditioning regimens and tailoring immunosuppressive strategies.^7^ This growing focus on immune reconstitution and TCR repertoire development not only emphasises the importance of αβ T‐cell recovery but also raises important questions about the restoration of other T‐cell subsets, including γδ+ T‐cells, which remain less well‐characterised following HSCT.

The reconstitution of T‐cell pools post‐HSCT occurs through two pathways: the thymic‐independent pathway, characterised by the homeostatic proliferation of T‐cells derived from the donor graft, and the thymic‐dependent pathway, in which donor‐derived progenitor stem cells give rise to naïve T‐cells in the thymus. Several transplant‐related factors, including conditioning regimen, GvHD prophylaxis^8^ and infections,^9^ are known to impact T‐cell reconstitution and influence TCR diversity.^2^, ^5^ While the reconstitution of αβ T‐cells has been extensively investigated,^2^, ^7^ fewer studies have elucidated how the γδ+ T‐cell compartment and TCR diversity are restored following HSCT.^10^

γδ+ T‐cells are uniquely equipped to respond rapidly to stress‐induced signals, acting fast in response to viral infections or malignant transformation. They achieve this through their distinct ability to recognise a diverse array of ligands, including phosphoantigens and stress‐induced ligands.^11^, ^12^ The HLA‐independence of γδ+ T‐cells is associated with a low risk of inducing GvHD.^13^, ^14^ The majority of circulating γδ+ T‐cells express the Vδ2 chain, commonly paired with the Vγ9 chain (Vδ2Vγ9), while a smaller subset expresses the Vδ1 chain. Both subsets have shown cytotoxicity against tumor cells, including AML.^15^, ^16^, ^17^, ^18^, ^19^ Increased γδ+ T‐cell numbers post‐HSCT are associated with improved overall survival, relapse‐free survival and decreased risk of infection.^14^, ^20^, ^21^, ^22^, ^23^, ^24^ γδ+ T‐cells reconstitute early after HSCT,^2^, ^5^, ^10^, ^25^, ^26^ with initial expansion occurring predominantly via peripheral proliferation,^2^, ^10^, ^14^, ^25^ often accompanied by repertoire skewing.^27^ *De novo* generation from haematopoietic stem cells was suggested from the observation of increasing naïve γδ+ T‐cell numbers post‐HSCT.^2^, ^18^ Notably, CMV reactivation was shown to influence γδ+ T‐cell expansion and repertoire skewing post‐HSCT.^18^, ^28^, ^29^ Despite their potential clinical relevance, few studies have systematically investigated γδ+ T‐cell phenotype and γδ TCR diversity in relation to clinical outcomes post‐HSCT.^10^ We therefore investigated the early reconstitution of γδ+ T‐cells in AML and myelodysplastic syndrome (MDS) patients undergoing allogeneic HSCT, characterising both on the TCR and on the phenotypic level.

## Results

### γδ+ T‐cells reconstitution dynamics reveal a transient reduction of TRG diversity post‐HSCT

We characterised the immune reconstitution, with a focus on γδ+ T‐cells, over a 12‐month (M) period post‐HSCT. All patients underwent HSCT for AML or MDS/myeloproliferative neoplasms (Table 1).

**Figure 1:**
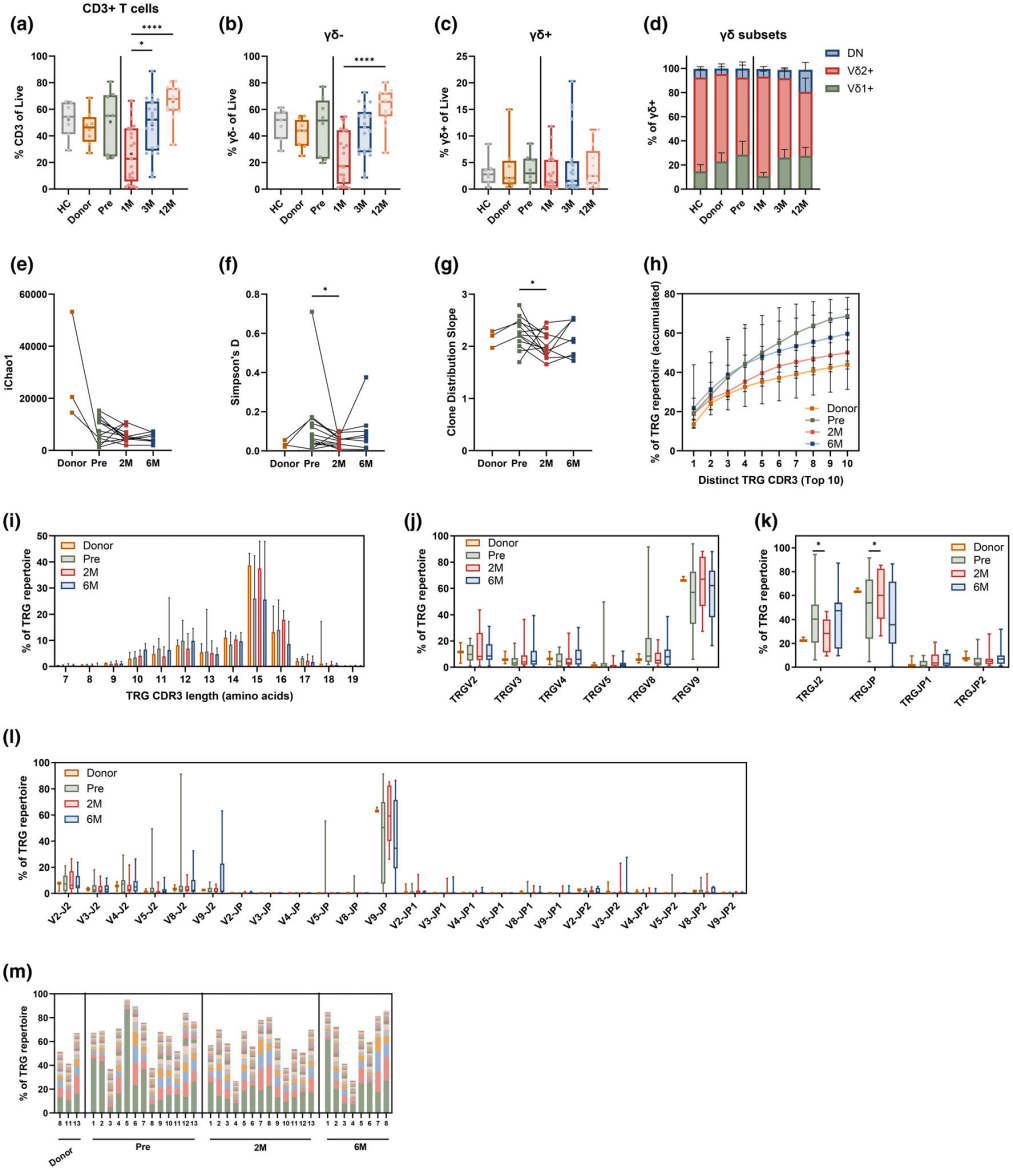
Overall γδ+ T‐cell phenotype early post‐HSCT. Frequency of CD3+ T‐cells **(a)**, γδ− T‐cells **(b)** and γδ+ T‐cells **(c)** of live PBMCs in controls (HC, donor and Pre‐HSCT) and at 1 M, 3 M and 12 M post‐HSCT. **(d)** Frequency of γδ subsets (Vδ1+, Vδ2+ and DN). TRG diversity as calculated by **(e)** iChao1 estimator, **(f)** Simpson's D index and **(g)** Clone distribution slope. **(h)** Cumulative frequency curves derived from the top 10 clonotypes. **(i)** CDR3 length distribution (in amino acids), displayed as the relative proportion of CDR3 sequences for each length weighed by the number of clones per clonotype. Median with IQR. **(j)** Frequency of TRG‐Variable segments usage. Range is shown. **(k)** Frequency of TRG‐Joining segments usage. Range is shown. **(l)** Frequency of the TRGV/J junctions. Range is shown. **(m)** Proportion of the top 20 clones among the TRG repertoire in each time point. Patients are numbered 1–13, and different clones within one patient are represented with different colours. **(a–c)** Kruskal–Wallis test with Dunn's multiple comparison, excluding HC. Range is shown. **(d)** Kruskal–Wallis test with Dunn's multiple comparison (not significant). Mean with SEM is shown. **(e–l)** Wilcoxon test comparing Pre vs 2 M. *P < 0.05, ****P < 0.0001. DN, double negative for Vδ1 and Vδ2; HC, healthy control; Pre, pre‐HSCT; TRG, T‐cell Receptor Gamma. 3 < N < 21.

The reconstitution of CD3+ T‐cells at 1 M, 3 M and 12 M post‐HSCT was analysed by FC (Supplementary figure 1) and compared to control samples, including the corresponding donor, the recipient before transplantation (pre) and healthy controls (HC). CD3+ T‐cell frequency was lowest at 1 M post‐HSCT (median of 20%) in comparison with all control groups (median > 40%, Figure 1a). The CD3+ T‐cell frequency increased from 1 M to 12 M, reaching similar levels to those observed in controls (Figure 1a). This recovery pattern could be attributed to the dominant γδ− (presumably αβ) T‐cell population, which followed a similar trajectory (Figure 1b; Supplementary figure 2a). In contrast, although γδ+ T‐cell frequencies varied widely between patients, they remained unchanged over time and did not differ significantly from controls at any time point (Figure 1c; Supplementary figure 2b). No significant changes were observed in the frequency of the different γδ+ T‐cell subsets over time, and high variability was observed between patients (Figure 1d; Supplementary figure 2c).

To monitor the γδ+ T‐cell reconstitution at the clonotypic level, the TRG CDR3 composition was assessed by NGS. Although no changes were observed in the iChao1 estimator (Figure 1e), both Simpson's D index and clone distribution slope were reduced at 2 M post‐HSCT as compared to pre‐HSCT, returning to basal levels at 6 M (Figure 1f and g). The clone distribution slope reflects the evenness of clonotype distribution, with a lower slope indicating increased dominance of a few expanded clones. These findings suggest a transient expansion of specific T‐cell clones at 2 M post‐HSCT, resulting in a temporary clonal dominance of a limited number of TRG clonotypes. Interestingly, this expansion had no impact on the top 10 most frequent sequences, as no significant differences were observed between the time points (Figure 1h). However, a trend towards a lower cumulative top 10 frequency at 2 M post‐HSCT could be observed, compared to pre‐HSCT.

The mean CDR3 length increased from 14 amino acids pre‐HSCT to 15 amino acids at 2 M, returning to basal levels at 6 M post‐HSCT, coinciding with the observed shift in diversity (Figure 1f, g and i; Supplementary figure 2d). TRGV9 was the most frequently used gene segment at all time points (Figure 1j). Given that Vδ2 typically pairs with Vγ9,^5^ this observation aligns with the FC data showing a dominant Vδ2+ proportion (Figure 1d). Moreover, the frequency of TRGJ2 gene segment usage increased from 2 M to 6 M post‐HSCT, while TRGJP usage decreased over the same period (Figure 1k). The TRGV9‐JP junction remained the most frequent across all time points (Figure 1l).

Analysis of the top 20 most abundant clones showed a relatively even distribution across time points, with high interpatient variability (Figure 1m). Altogether, this suggests that γδ+ T‐cell frequency is already stable at early time points post‐HSCT and their clonotypic composition is transiently altered at 2 M post‐HSCT. This change is associated with the expansion of less frequent TRGV9‐JP clones featuring longer CDR3 regions.

### γδ+ T‐cell subsets display distinct differentiation phenotypes

**Figure 2:**
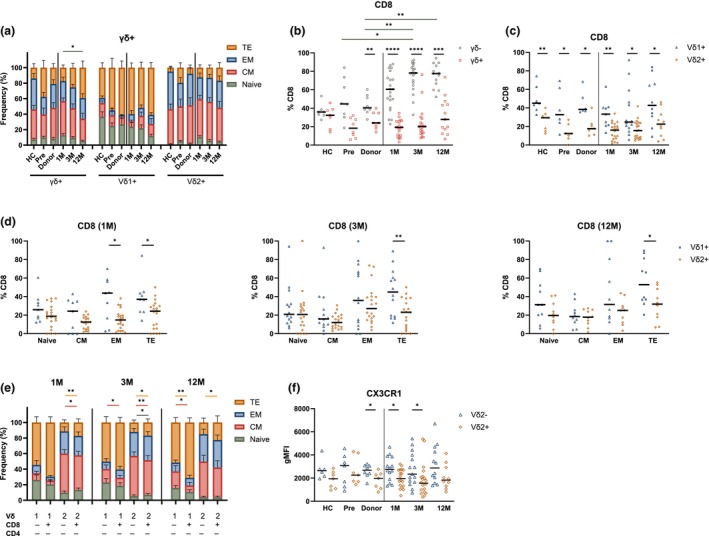
Effector phenotype of γδ+ T‐cells post‐HSCT. **(a)** Proportion of differentiation subsets in γδ+, Vδ1+ and Vδ2+ T‐cells. **(b–d)** Comparison of CD8 frequency between γδ− and γδ+ T‐cells **(b)**, Vδ1+ and Vδ2+ T‐cells **(c)** and within differentiation T‐cell subsets of Vδ1+ and Vδ2+ T‐cells at 1 M, 3 M and 12 M post‐HSCT **(d)**. **(e)** Proportion of differentiation subsets in Vδ1+ and Vδ2+, CD4−CD8− and CD4−CD8+ cells. **(f)** Comparison of CX3CR1 expression (gMFI) between Vδ2− and Vδ2+ T‐cells. **(a, b)** Kruskal–Wallis test to compare time points, excluding HC. **(b)** Wilcoxon test to compare cell populations at each time point. **(c, d, f)** Mann–Whitney test to compare cell populations at each time point. **(e)** Wilcoxon test to compare CD4‐CD8‐ and CD4‐CD8+. **(a, e)** Mean with SEM is shown. **(b, c, d, f)** Median is shown. *P < 0.05, **P < 0.01, ***P < 0.001, ****P < 0.0001. CM, central memory (CD27+ CD45RO+); EM, effector memory (CD27− CD45RO+); gMFI, geometric mean fluorescence intensity; HC, healthy control; Pre, pre‐HSCT; TE, terminal effector (CD27− CD45RO−). 6 < N < 21.

We next examined the γδ+ T‐cell differentiation state (Supplementary figure 3a). Overall, γδ+ T‐cells exhibited a predominant central memory (CM) phenotype, with a progressive increase in terminal effector (TE) phenotype over time post‐HSCT (1 M vs. 12 M, Figure 2a). Interestingly, Vδ2+ T‐cells maintained a CM phenotype (> 40%, Figure 2a), while Vδ1+ T‐cells were mainly of TE phenotype (> 40%), and showed significantly increased  TE and reduced naive subsets in comparison to HC (Figure 2a; Supplementary figure 3b). In contrast to γδ+ T‐cells, γδ− T‐cells were predominantly of effector memory (EM) phenotype (Supplementary figure 3c, d).

We next compared the CD8 frequency between T‐cell subsets. As expected, γδ− T‐cells exhibited the highest frequency of CD8+ T‐cells, especially post‐HSCT in comparison to donor and pre‐HSCT samples (Figure 2b). Within the γδ+ compartment, CD8 frequency was higher in Vδ1+ than in Vδ2+ T‐cells across both controls and post‐HSCT samples (Figure 2c). This trend was particularly pronounced among Vδ1+ T‐cells with an effector phenotype, especially those with a TE phenotype, which exhibited a consistently higher CD8+ frequency than Vδ2+ T‐cells post‐HSCT (Figure 2d). Similar trends could be observed in the controls (Supplementary figure 3e). When comparing differentiation phenotypes within CD4−CD8+ and CD4−CD8− γδ+ T‐cell subsets, we also observed that Vδ2+ CD4−CD8+ cells had a higher TE proportion than Vδ2+ CD4−CD8− cells. Within Vδ1+, this difference was only significant at 12 M (Figure 2e).

To further investigate their differentiation state, we assessed the CX3CR1 expression, a marker associated with cytotoxicity and tissue migration.^30^, ^31^ CX3CR1 expression was higher in γδ+ T‐cells than in γδ− T‐cells (Supplementary figure 3f). Additionally, Vδ2− T‐cells expressed higher levels of CX3CR1 than Vδ2+ T‐cells in donor samples at 1 M and 3 M post‐HSCT (Figure 2f), consistent with the higher TE content observed in Vδ1+ cells (Figure 2a).

### γδ+ T‐cell subsets display distinct inhibitory and NK‐like phenotypes

**Figure 3:**
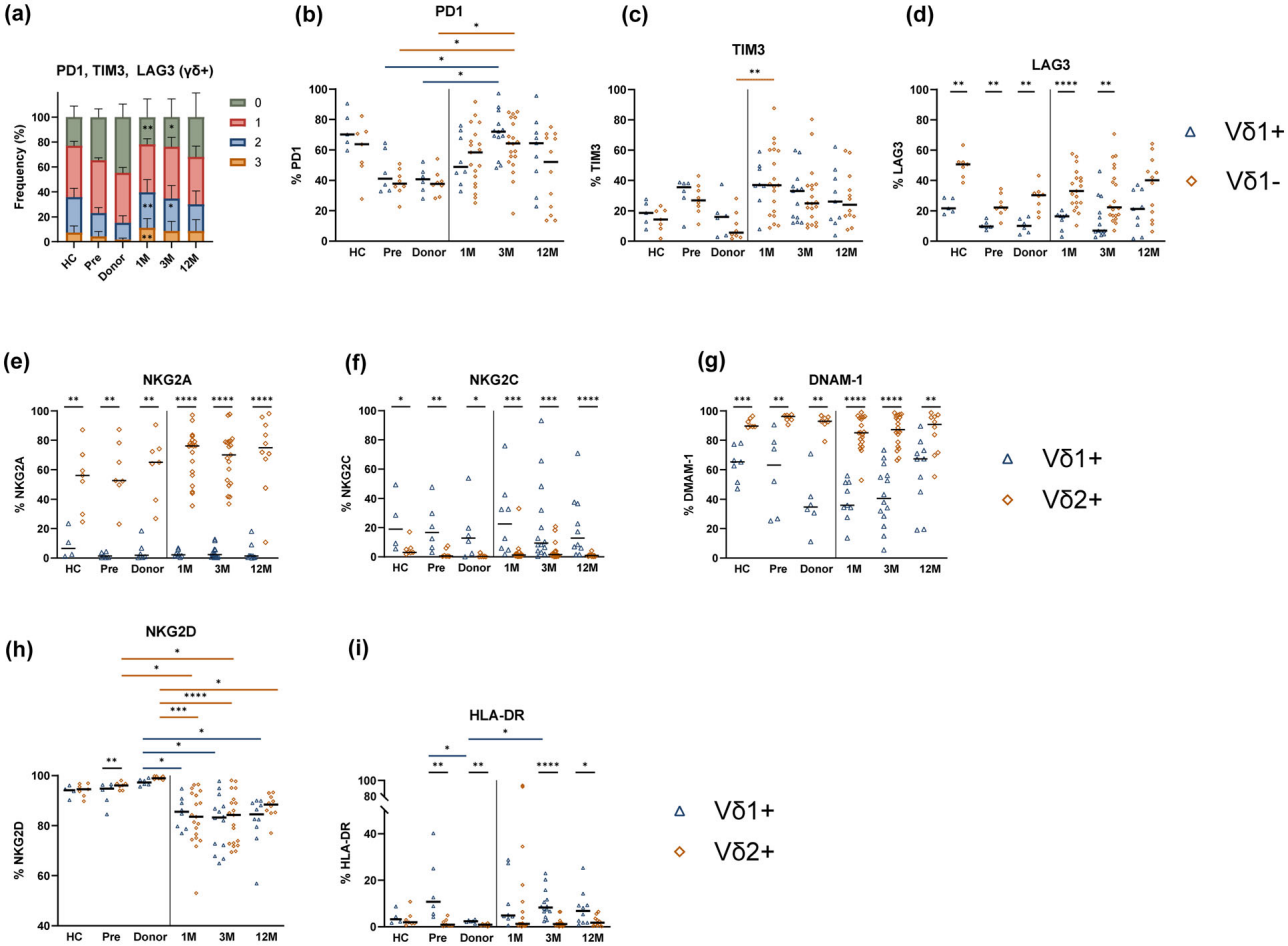
Characterisation of inhibitory and γδ+ T‐cell‐specific phenotype post‐HSCT. **(a)** Bar chart representing the number of co‐expression of PD1, TIM3 and LAG3 in γδ+ T‐cells. Mean with SEM is shown. **(b–j)** Comparison of frequency of PD1 **(b)**, TIM3 **(c)**, LAG3 **(d)**, NKG2A **(e)**, NKG2C **(f)**, DNAM‐1 **(g)**, NKG2D **(h)** and HLA‐DR **(i)** expressing Vδ1+ and Vδ2+ (or Vδ1‐) T‐cells. **(a)** Kruskal–Wallis test with Dunn's multiple comparisons between indicated post‐HSCT time points and donors. **(b–i)** Mann–Whitney test (Vδ1+ vs Vδ2+ T‐cells) and Kruskal–Wallis test (between time points). Median is shown. *P < 0.05, **P < 0.01, ***P < 0.001, ****P < 0.0001. HC, healthy control; Pre, pre‐HSCT. 6 < N < 21.

We then investigated the expression of the immune checkpoints (ICs) PD‐1, TIM‐3 and LAG‐3. Peaks in TIM‐3 and PD‐1 frequency were observed at 1 M and 3 M post‐HSCT, respectively, while LAG‐3 frequency remained stable over time (Supplementary figure 4a). Overall, γδ+ T‐cells showed higher frequencies of LAG3 and TIM3 than γδ− T‐cells, both in controls and at post‐HSCT time points (Supplementary figure 4a). In γδ+ T‐cells, distinct patterns of IC co‐expression were observed between early time points post‐HSCT and donor samples. Up to 3 M post‐HSCT, γδ+ T‐cells exhibited a higher frequency of triple‐ and double‐positive IC expression and a reduced frequency of triple‐negative IC expression compared to donors (Figure 3a; Supplementary figure 4b). This co‐expression pattern was even more distinct in γδ− T‐cells, where a significant decrease in double‐ and triple‐positive cells from 1 M to 12 M was observed, suggesting a gradual recovery from an exhaustive phenotype over time post‐HSCT (Supplementary figure 4c). Within γδ T‐cell subsets, no major changes were observed in IC frequency kinetics (Figure 3b–d). However, a higher frequency of PD‐1+ γδ+ T‐cells was noted at 3 M in comparison with the pre‐HSCT and donor groups, and LAG‐3 frequency was consistently higher in Vδ1− T‐cells than in Vδ1+ T‐cells (Figure 3d).

We further investigated phenotypic differences between Vδ1+ and Vδ2+ T‐cells. As expected, Vδ1+ T‐cells displayed a higher frequency of NKG2C and lower NKG2A and DNAM‐1 frequencies than Vδ2+ T‐cells across all sample groups (Figure 3e–g). These markers remained stable over time post‐HSCT within the cellular subsets. In contrast, NKG2D frequency was reduced post‐HSCT as compared to controls (Figure 3h). In parallel, the activation marker HLA‐DR was more frequently expressed in Vδ1+ than in Vδ2+ T‐cells in pre‐HSCT, donor, 3 M, and 12 M samples (Figure 3i). CD28 frequency did not differ significantly across γδ+ T‐cell subsets (Supplementary figure 4d), while a lower frequency of CD69+ cells post‐HSCT was observed compared to controls (Supplementary figure 4e).

Overall, γδ+ T‐cells showed signs of exhaustion early post‐HSCT but recovered over time post‐HSCT, and the NKG2D frequency on γδ+ T‐cells appeared to be specifically affected by HSCT.

### Public TRGV9 and private non‐TRGV9 clonotypes contribute to γδ+ T‐cell reconstitution

**Figure 4:**
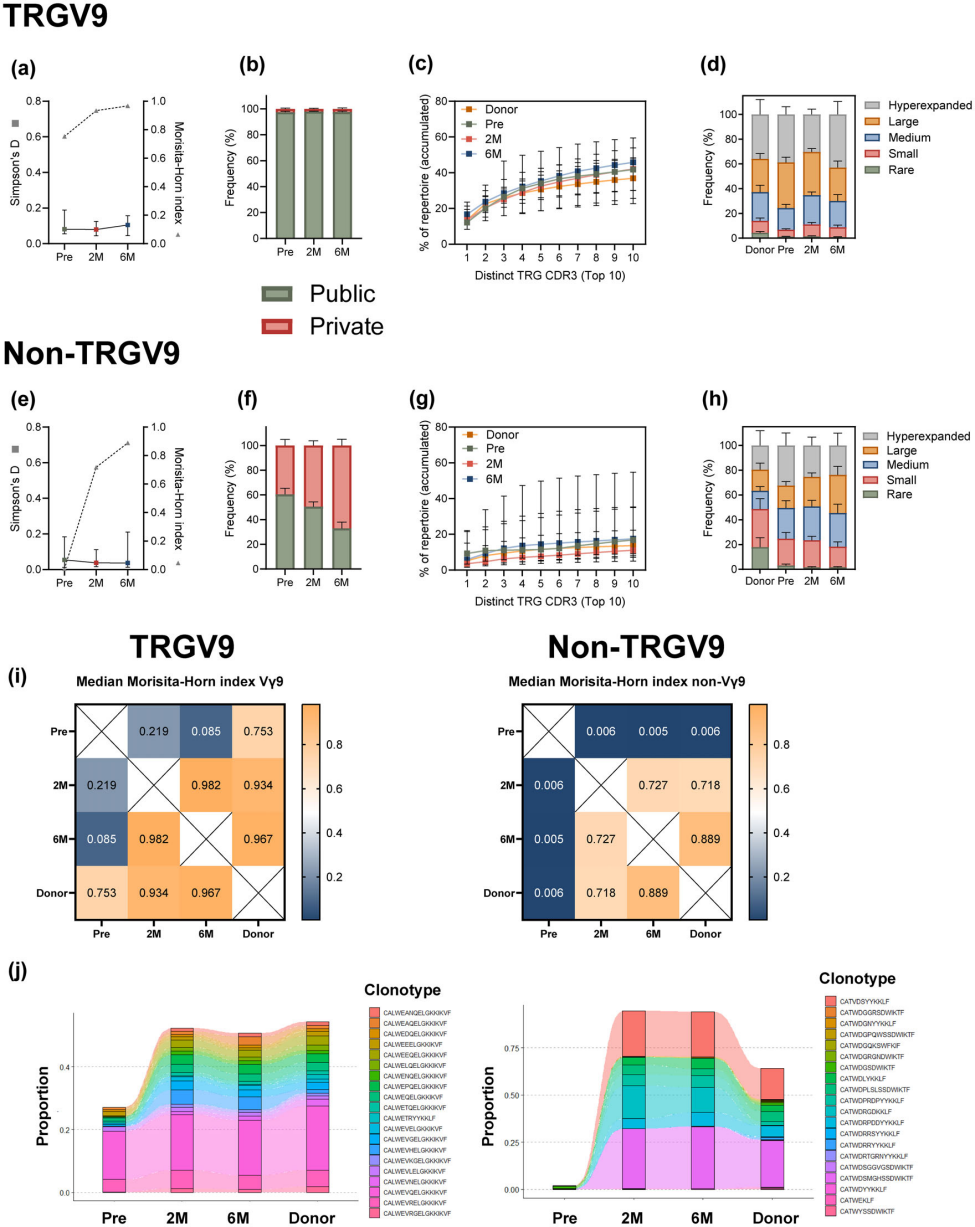
TRGV9 and non‐TRGV9 clonotypes reconstitution. TRGV9 **(a)** and non‐TRGV9 **(e)** clonotypes diversity as calculated by Simpson's D index (■) and donor–recipient TCR overlap using the Morisita–Horn Index (▲). Median of three donor–recipient pairs is shown. Frequency of public (found in > 1 donor) and private (found in only one donor) TRGV9 **(b)** and non‐TRGV9 **(f)** clonotypes. Mean with SEM is shown. Cumulative frequency curves derived from the top 10 TRGV9 **(c)** and non‐TRGV9 **(g)** clonotypes in each time point. Median with IQR is shown. Homeostatic space occupied by TRGV9 **(d)** and non‐TRGV9 **(h)** clonotypes classified as hyperexpanded (> 1%), large (0.1–1%), medium (0.01–0.1%), small (0.001–0.01%), and rare (0–0.001%). Mean with SEM is shown. **(i)** Median of Morisita–Horn index in TRGV9 (left) and non‐TRGV9 (right) clonotypes. **(j)** Tracking of donor top 20 TRGV9 (left) and non‐TRGV9 (right) clonotypes at different time points in one representative patient. Pre, pre‐HSCT. 3 < N < 13.

TRGV9 is the most commonly used gene segment in peripheral blood^10^ and graft γδ T‐cells.^32^ As we observed a high correlation between the expression patterns of Vδ2+ and Vγ9+ cells by FC (Supplementary figure 5a), we assessed the contribution of TRGV9 clonotypes to early reconstitution following HSCT as compared to non‐TRGV9 clones. The frequency of TRGV9 clonotypes remained unchanged over time (Supplementary figure 5b), matching FC data (Supplementary figure 5c). Although TRGV9 repertoire diversity did not significantly change post‐HSCT, the median overlap with the donor's TCR repertoire increased post‐HSCT, indicating a growing contribution of donor‐derived clonotypes (Figure 4a and i). The TRGV9 clonotypes remained predominantly public (Figure 4b), and the distribution of the top 10 most frequent clonotypes was similar between pre‐ and post‐HSCT time points (Figure 4c). No striking difference was observed in the homeostatic space occupied by TRGV9 clonotypes (Figure 4d).

In contrast, non‐TRGV9 clonotypes showed low repertoire overlap with the donor repertoire pre‐HSCT, which increased post‐HSCT, despite no significant changes in the TCR diversity (Figure 4e and i). These clonotypes were mostly shorter and public pre‐HSCT but evolved into a more private profile over time post‐HSCT (Supplementary figure 5d, Figure 4f). No difference was observed in the frequency of the top 10 clonotypes between time points (Figure 4g), although a mild expansion of larger clonotypes was noted post‐HSCT (Figure 4h). Overall, TRGV9 clonotypes showed greater overlap across time points than non‐TRGV9 clonotypes, with the highest overlap occurring between 2 M and 6 M post‐HSCT (Figure 4i).

To map the clonotypes' origin, we tracked the top 20 CDR3 amino acid sequences at 6 M post‐HSCT. For TRGV9, these dominant clonotypes were already present pre‐HSCT, expanded at 2 M, and matched donor levels at 6 M (Figure 4j, left). In contrast, the top 20 non‐TRGV9 clonotypes were rare pre‐HSCT, expanded at 2 M, and remained nearly identical at donor levels at 6 M (Figure 4j, right).

Altogether, these findings suggest that the early reconstitution of γδ T‐cells post‐HSCT is driven by the homeostatic expansion of residual and public TRGV9 clonotypes that are shared with the donor, alongside the emergence and expansion of donor‐derived private non‐TRGV9 clonotypes.

### γδ+ T‐cells show CD8+ association with acute GVHD and reduced sensitivity to anti‐thymocyte globulin (ATG)

As we previously reported an association between CD8+ γδ+ T‐cells in grafts and the development of acute GVHD (aGVHD),^33^ we examined whether post‐HSCT γδ+ T‐cells were associated with aGVHD. Overall, we found no association between aGVHD development and the frequency of total γδ+ T‐cells, γδ+ T‐cell subsets and TRGV9 usage (Supplementary figure 6a, b). However, at 3 M post‐HSCT, we observed a higher frequency of CD8+ γδ+ T‐cells in the aGVHD grade II–III group (Supplementary figure 6c). No other (i.e. TRG repertoire or γδ+ and γδ− T‐cell phenotype) significant association with aGVHD was found (Supplementary figure 6d–f).

**Figure 5:**
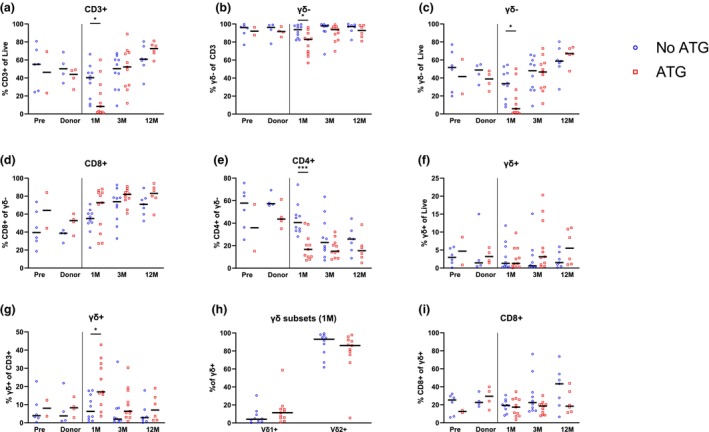
ATG treatment affects γδ− T‐cells more than γδ+ T‐cells. Frequency of CD3+ T‐cells among live cells **(a)**, γδ− T‐cells among CD3+ T‐cells **(b)**, γδ− T‐cells among live cells **(c)** in patients with or without ATG treatment. Frequency of CD8+ **(d)** and CD4+ **(e)** cells among γδ − T‐cells in patients with or without ATG treatment. Frequency of γδ+ T‐cells among live cells **(f)** and among CD3+ T‐cells **(g)** in patients with or without ATG treatment. Frequency of **(h)** Vδ1+ and Vδ2+ subsets among γδ+ T‐cells at 1 M post‐HSCT and **(i)** CD8+ cells among γδ+ T‐cells in patients with or without ATG treatment. **(a‐i)** Mann–Whitney test to compare patient groups. Medians are represented. *P < 0.05.

To further decipher the association between CD8+ γδ+ T‐cells and aGVHD, we grouped the patients by their GVHD prophylaxis, that is ATG. ATG treatment reduced the overall percentage of CD3+ T‐cells at 1 M post‐HSCT (Figure 5a) but, interestingly, this seemed to only affect γδ− T‐cells (Figure 5b and c), in particular the CD4+ γδ− T‐cells subset (Figure 5d and e), while the total γδ+ T‐cell percentage remained unchanged (Figure 5f). This led to an increased γδ+ T‐cell percentage among CD3+ in the ATG‐treated patient's group (Figure 5g). Frequencies of Vδ1+ and Vδ2+ were not affected by ATG prophylaxis as shown at 1 M (Figure 5h), and neither was the GVHD‐relevant CD8 frequency among γδ+ T‐cells (Figure 5i).

Altogether, this suggests that γδ+ T‐cells may have a reduced sensitivity to ATG‐mediated depletion compared to other T‐cell subsets such as αβ T‐cells. The higher frequency of CD8 in γδ+ T‐cells observed in the aGHVD cohort does not appear to be influenced by the ATG prophylaxis.

### γδ+ T‐cell differentiation phenotype is associated with CMV reactivation

**Figure 6:**
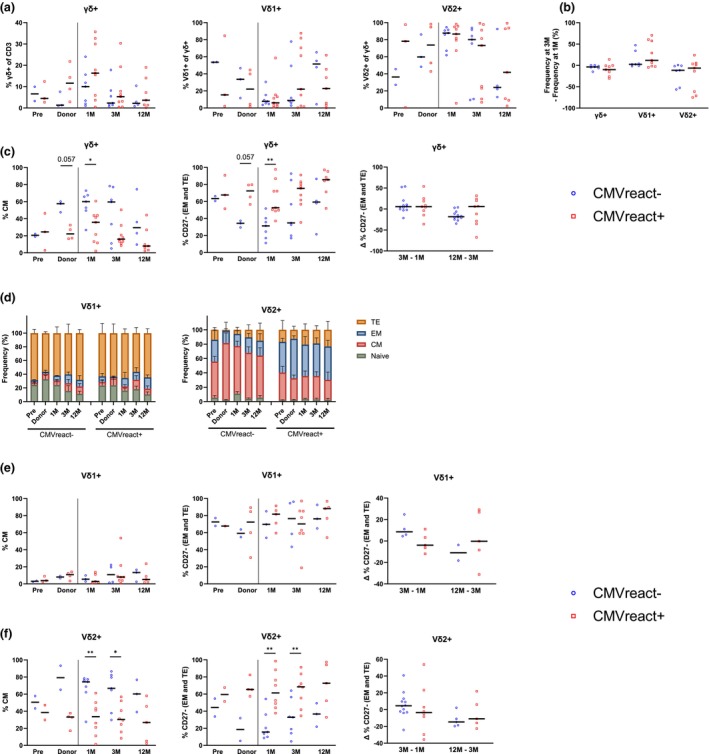
CMV reactivation is associated with a distinct γδ+ T‐cell phenotype. **(a)** Frequency of γδ+, Vδ1+ and Vδ2+ T‐cells in patients with or without CMV reactivation. **(b)** Frequency of γδ+, Vδ1+ and Vδ2+ T‐cells at 3 M – frequency at 1 M. **(c)** Frequency of CM γδ+ T‐cells (left), of CD27− (EM/TE) γδ+ T‐cells (middle), and of CD27− γδ+ T‐cells at 3 M–1 M and 12 M–3 M (right). **(d)** Proportion of differentiation states in Vδ1+ and Vδ2+ T‐cells upon CMV reactivation. Mean with SEM. Frequency of CM (left), CD27− (EM/TE, middle), and frequency of CD27− at 3 M–1 M and 12 M–3 M (right) within Vδ1+ **(e)** and Vδ2+ **(f)** T‐cells in patients with or without CMV reactivation. All graphs exclude D−R− CMV− patients. **(a, b, c, e, f)** Mann–Whitney test to compare patient groups. Medians are represented. *P < 0.05, **P < 0.01. CM, central memory; EM, effector memory; HC, healthy control; Pre, Pre‐HSCT; TE, terminal effector. 3 < N < 12.

γδ+ T‐cells contribute to viral control in immunocompromised patients; therefore, we investigated how CMV reactivation post‐HSCT influenced γδ+ T‐cells phenotype and clonality. No association was observed between CMV reactivation or CMV serostatus and frequencies of total γδ+ T‐cells or subsets over time post‐HSCT, although a trend towards higher Vδ1+ cell frequency could be observed in CMV‐seropositive recipients (R+) and their donors (D− or D+) (Figure 6a and b; Supplementary figure 7a–c). This trend was supported by NGS data showing that pre‐HSCT, R+ patients who experienced CMV reactivation post‐HSCT (CMVreact+) exhibited an increased non‐TRGV9 frequency in the γδ T‐cell repertoire compared to R‐CMVreact− patients. At 2 M post‐HSCT, this difference was only visible between R−CMVreact− and R+CMVreact−, pointing towards a role of patient pre‐transplant CMV serostatus on γδ T‐cell subsets (Supplementary figure 7d).

However, in CMVreact+ patients, γδ+ T‐cells showed a reduced proportion of CM phenotype and an increased proportion of effector phenotypes (CD27−; EM, TE) at 1 M compared to CMVreact− patients (of which D−R− patients were excluded) (Figure 6c; Supplementary figure 7e). Of note, this trend was also observed in the donor samples, and no change was observed over time post‐HSCT (Figure 6c). The difference between CMV groups was most pronounced within the Vδ2+ T‐cell subset (Figure 6d–f). This suggests that a CM phenotype in donor‐derived Vδ2+ T‐cells may be protective, whereas a higher proportion of effector phenotypes may increase the risk of CMV reactivation (median of 39 days, Table 1).

In contrast, γδ− T‐cells in CMVreact+ patients exhibited no difference in central or effector memory cells and increased CD8 frequency at 6 M post‐HSCT (Supplementary figure 8a, b). Regarding IC co‐expression, CMV reactivation was associated with fewer triple‐negative γδ− T‐cells at 1 M, while no association was observed at other time points or in γδ+ T‐cells (Supplementary figure 8c).

Altogether, these data suggest distinct associations of CMV reactivation with γδ− and γδ+ T‐cell phenotypes, especially with the differentiation state—possibly of donor origin—of Vδ2+ γδ T‐cells.

### Association of CMV reactivation with pre‐HSCT γδ+ T‐cell clonality and phenotype

**Figure 7:**
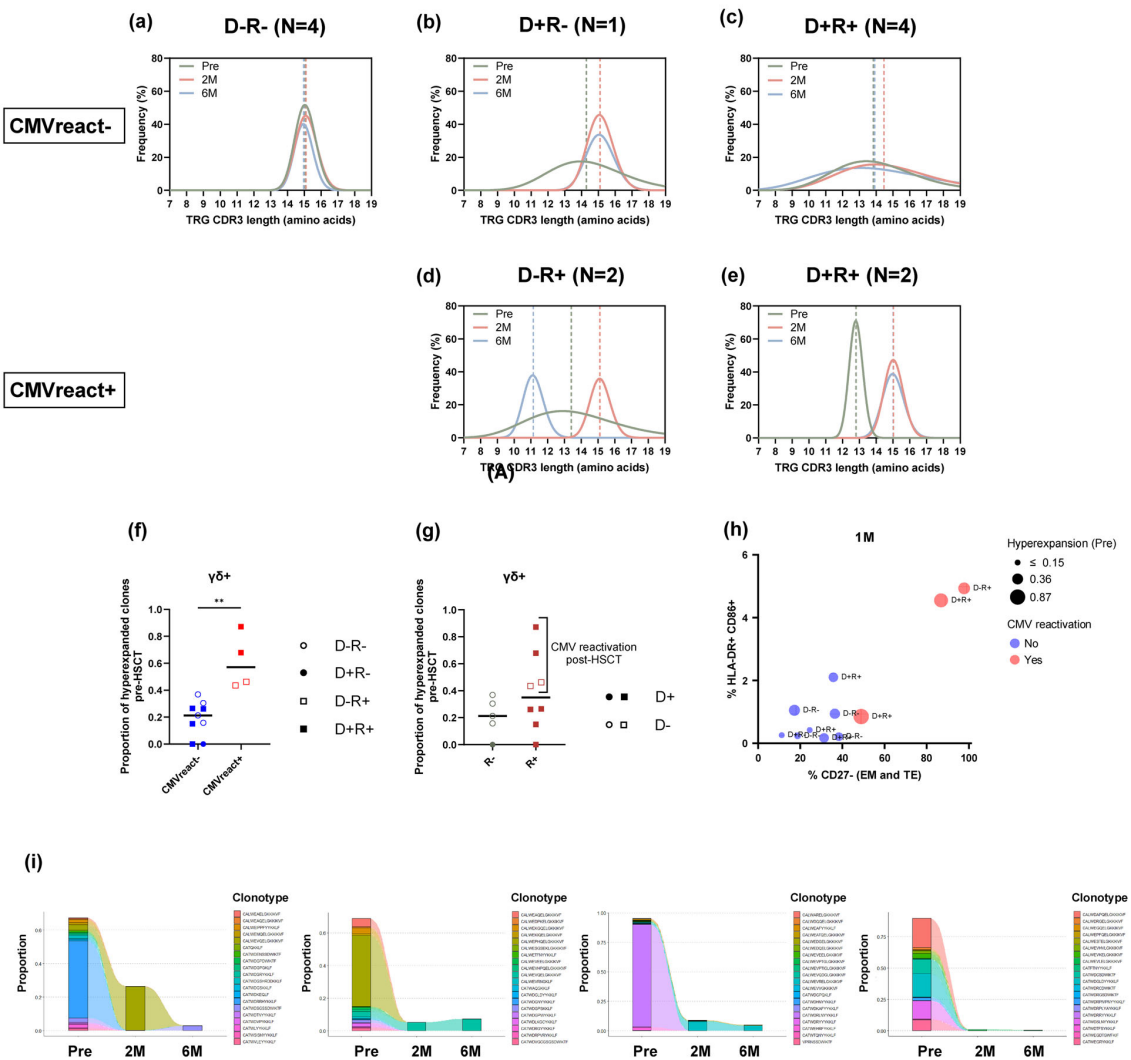
CMV reactivation is associated with expansion of HLA‐DR+ CD86+ γδ+ T‐cells. CDR3 length distribution (in amino acids), displayed as the relative proportion of CDR3 sequences for each length weighed by the number of clones per clonotype for patients who did not experience CMV reactivation (CMVreact−) with a D−R− **(a)**, D+R− **(b)**, and D+R+ **(c)** serostatus, and patients who experienced CMV reactivation (CMVreact+) with a D−R+ **(d)** and D+R+ **(e)** serostatus. The curves represent nonlinear curve fitting (Gauss function) applied to the frequency of TRG lengths in each group. Vertical lines denote the mean CDR3 length for each group. Frequency of hyperexpanded γδ clones pre‐HSCT by CMV reactivation status post‐HSCT **(f)**, and by recipient CMV‐serostatus **(g)**. **(h)** Association between HLA‐DR and CD86 co‐expression at 1 M post‐HSCT (y‐axis), CD27− frequency (x‐axis), CMV reactivation status (colour), proportion of clone hyperexpansion (circle size) pre‐HSCT and serostatus (label) in γδ+ T‐cells. **(i)** Tracking of top 20 clonotypes in CMVreact+ patients with hyperexpanded pre‐HSCT clones, at post‐HSCT time points. These patients all received RIC. **(f–g)** Mann–Whitney test to compare groups, excluding D−R− patients from the CMVreact− group. Medians are shown. 3 < N < 9.

As we observed differences in γδ+ T‐cell subset frequency and phenotype by CMV‐serostatus and reactivation, we also investigated the spectratype of these groups: Interestingly, while no changes were observed over time, spectratypes of CMVreact− patients were focussed around 15 aa for D−R− (Figure 7a) and D+R− (Figure 7b) serostatus, while they were broader and closer to 14 aa for D+R+ serostatus (Figure 7c), indicating a possible impact of CMV presence on CDR3 length. Similarly, all CMVreact+ patients were R+ and their pre‐HSCT lengths were shorter (around 13 aa) (Figure 7d and e, green lines). Interestingly, their donor seemed to affect their spectratypes particularly at 6 M, with D− causing a drastic shortening of CDR3 length down to 11 aa (Figure 7d), while D+ led to unchanged spectratypes of the recipient, around 15aa (Figure 7e). This suggests that the encounter of CMV for the first time (such as when D− cells encounter an R+ host) may be associated with shorter CDR3. Moreover, we observed that CMVreact+ patients displayed a high proportion of hyperexpanded γδ TCR clones pre‐HSCT in comparison with CMVreact− patients (Figure 7f). CMV reactivation occurred exclusively in CMV‐seropositive patients (R+); yet, serostatus alone did not account for the increased frequency of hyperexpanded γδ clonotypes pre‐HSCT (Figure 7g). CMVreact+ patients with a high proportion of hyperexpanded γδ+ T‐cell clones pre‐HSCT also showed higher HLA‐DR and CD86 co‐expression and CD27− frequency at 1 M post‐HSCT (Figure 7h) with a strong correlation between HLA−DR+ and CD27− cells (Supplementary figure 8d). Last, tracking the top 20 pre‐HSCT clonotypes revealed that many were lost early post‐HSCT despite the reduced intensity conditioning (RIC: 9/10 CMVreact+ patients, Table 1, Figure 7i). When tracking the top 20 clonotypes present at 6 M, we did not observe consistent alterations in CMVreact+ patients compared to CMVreact− patients (Supplementary figure 9).

## Discussion

A comprehensive characterisation of the γδ+ T‐cell repertoire and its early reconstitution dynamics post‐HSCT remains insufficiently explored. In this study, we longitudinally profiled the γδ+ T‐cell repertoire and phenotype post‐HSCT and observed that the phenotypical differences observed between Vδ1+ and Vδ2+ cell subsets in healthy and pre‐HSCT were conserved post‐HSCT. This demonstrates that even after a major immunological reset event such as HSCT, the fundamental signatures of Vδ1+ and Vδ2+ T‐cells remain unchanged over time. In addition, the TRG CDR3 repertoire dynamics progressively normalised by 6 M post‐HSCT, with the repertoire resembling pre‐HSCT diversity levels, top clones' frequencies, CDR3 length distributions and TRGV/TRGJ gene pairings, including an enrichment of V9‐JP usage consistent with healthy individuals and cord blood. This pairing is reactive to phosphoantigens and associated with innate‐like Vγ9Vδ2 cells.^10^

In general, the overall phenotype of γδ+ T‐cells post‐HSCT was comparable to that of controls. Frequencies of total γδ+ T‐cells, Vδ1/Vδ2 proportion, the expression of key phenotypic markers as well as differentiation phenotype dominated by a CM profile, were comparable. Similarly, expression levels of IC markers were largely consistent with controls, despite some temporal evolution post‐HSCT. Only NKG2D frequency was significantly lower on γδ+ T‐cells post‐HSCT. Since NKG2D mediates recognition of stress‐induced ligands via MICA/MICB, this reduction may compromise γδ+ T‐cells' antileukemic potential.^34^

Our data emphasises the divergent dynamics between TRGV9 and non‐TRGV9 repertoires post‐HSCT.^29^, ^35^ TRGV9 (mostly public) clonotypes showed high donor–recipient overlap and stability over time, while non‐TRGV9 repertoires (more private) exhibited low pre‐HSCT overlap with donor repertoires and progressively increased overlap post‐HSCT, suggesting effective engraftment and expansion of donor‐derived private clonotypes. These observations are consistent with prior studies showing the public nature of Vδ2+/TRGV9 TCRs^29^ and the private, individual‐specific nature of Vδ1+/non‐TRGV9 TCRs.^35^ Supporting this, Ravens et al.^10^ also reported that TRG repertoires remain unchanged over time in both healthy adults and early post‐HSCT. This data suggests efficient conservation of donor‐derived clonotypes and their abundance in HSCT recipients.

ATG is used to deplete T‐cells and prevent GVHD. Here, we observed no effect of ATG on γδ T‐cells. Previous studies are conflicting in that regard: Klyuchnikov et al. showed that patients treated with ATG presented a higher γδ+ T‐cell count,^23^ while others showed no quantitative difference between different doses of ATG.^36^ We showed an increased γδ+ T‐cell frequency among CD3+ cells in ATG‐treated patients that is possibly associated with an ATG‐dependent decrease in the absolute number of αβ+ T‐cells. Whether γδ+ T‐cells are fully resistant to ATG or merely less affected than αβ+ T‐cells warrants further investigation.

γδ+ T‐cell expansion and differentiation state have been strongly associated with CMV reactivation, driving their proliferation, skewing towards an effector phenotype and altering the repertoire of Vδ1+ T‐cells.^10^, ^14^, ^18^, ^28^, ^37^, ^38^ In our cohort, CMV reactivation occurred only in CMV seropositive patients (R+) and was associated with a dominant effector phenotype of Vδ2+ γδ T‐cells, also marked by increased HLA‐DR and CD86 co‐expression. This could suggest a possible cytotoxic role in controlling CMV infection as previously described.^18^ Yet, in our study, we did not observe an increased proportion of this effector phenotype after the time point of CMV reactivation. Instead, CMVreact+ patients presented a lower proportion of CM γδ+ T cells than CMVreact‐ patients early post‐HSCT, before the time point of CMV reactivation, and the same difference was observed in their respective donors. This raises the possibility that having a high proportion of donor‐derived CM γδ+ T‐cells may confer some direct or indirect protective effect against CMV reactivation post‐HSCT. CM cells, especially CD8+, have previously been reported to provide effective antitumor and anti‐CMV immunity,^39^, ^40^, ^41^ while CMV‐specific CM T cells with poor recovery early post‐HSCT—most probably of donor origin—have been associated with refractory CMV reactivation.^42^ More recently, the protective role of murine CMV‐induced γδ T cells was also described to rely on the presence of CM γδ+ T‐cells.^43^ To our knowledge, this association has not previously been reported in humans in a HSCT setting. Further investigations of γδ+ T‐cell memory phenotype pre‐ and post‐HSCT in the context of CMV reactivation in a larger cohort would be valuable.

Despite previous reports that CMV reactivation can reshape the TRG repertoire,^10^, ^32^ we did not observe consistent repertoire alterations using clonotype tracking. However, we observed that the four CMVreact+ patients exhibited a disturbed spectratype over time and a higher proportion of hyperexpanded γδ TCR clones in the pre‐HSCT samples. We also noted that those pre‐HSCT hyperexpanded clones were depleted despite the RIC regimen as demonstrated by the chimerism and clonotype tracking pre‐ and post‐HSCT (2 M). Altogether, one can speculate that some of these hyperexpanded clones were providing CMV control, and that their depletion under conditioning may have contributed to the CMV reactivation. Yet it is not possible to solely attribute the depletion of hyperexpanded γδ TCR clones to the occurrence of CMV reactivation since the clonality of conventional T‐cells was not investigated in our study.

The limitations of our study include a small cohort size and missing samples at certain time points, particularly for donors and pre‐HSCT, which reduced the statistical power of our findings. This was especially apparent when patients were grouped based on clinical events, such as aGVHD and CMV reactivation. Additionally, the limited number of γδ+ T‐cells per sample prevented us from evaluating subsets like Vδ1‐Vδ2‐, and from performing TRG CDR3 sequencing, γδ+ T‐cell phenotyping at matching time points, and functional assays to evaluate the cytotoxic role of γδ+ T‐cells in CMV infection. Lastly, our study included only patients who were alive at 12 months post‐HSCT, which may bias the analysis by the exclusion of patients with poor outcomes.

In this study, the combined phenotypical analysis with matched TCR sequencing of γδ+ T‐cells in HSCT provides important insight into their reconstitution and association with CMV reactivation. These results could pave the way for more extensive *in vitro* studies of a potential protective role of CM γδ+ T‐cells pre‐HSCT in CMV reactivation post‐HSCT. Future studies investigating the diversity of the gamma and delta chains of HSCT donors and recipients at a single‐cell level could further elucidate their role in the outcome of HSCT.

## Patients and methods

### Donor and patient characteristics

All patients underwent allogeneic HSCT at Karolinska University Hospital, Sweden, for a period of 24 months. Included patients were alive after 12 months post‐HSCT. Informed consent was obtained from patients and their corresponding donors, and ethics approval was obtained from the Swedish Ethical Review Authority (Stockholm) according to the Declaration of Helsinki. Peripheral blood (PB) samples were collected before conditioning and at 1, 2, 3, 6 and 12 months post‐HSCT. Matched donor PB graft samples were also obtained. Grading of GvHD was performed as previously described.^44^, ^45^ CMV was monitored by CMV‐PCR on plasma samples once a week after engraftment until 3 months post‐HSCT. Reactivation was defined as detectable CMV > 2000 IU/mL. No CMV‐specific prophylaxis was given, and pre‐emptive treatment with valganciclovir (900 mg twice a day) was given upon reactivation. Patient characteristics are summarised in Table 1 and clinical samples used in the study are summarised in Supplementary table 1. Seven healthy anonymised buffy coats were included as controls.

**Table 1:** Patient characteristics

Characteristics	Patients
Total number	22
Gender: female/male, *n*	7/15
Donor gender female/male, *n*	6/16
Gender match, yes/no, *n*	15/7
If gender mismatch: F → M/M → F, *n*	3/4
Recipient Age: median (range), years	63 (20–70)
Donor Age: median (range), years	30 (20–65)
Recipient CMV serostatus pre‐HSCT, −/+, *n*	6/16
Donor CMV serostatus pre‐HSCT, −/+, *n*	10/12
CMV serostatus (experienced CMV reactivation)	
D+/R+	11 (6)
D+/R−	1 (0)
D−/R+	5 (4)
D−/R−	5 (0)
Donor type, *n*	
HLA identical sibling	7
Haploidentical	2
HLA‐MUD	11
Allelic mismatched unrelated donor	2
Diagnosis, *n*	
AML	18
MDS / MPS	4
Conditioning regimen, *n*	
Myeloablative (MAC)/reduced intensity (RIC)	5 / 17
Busulphan + cyclophosphamide	5
Fludarabine + treosulfan	9
Fludarabine + busulfan (8 mg/kg)	7
N/A	1
Graft source, *n*	
BM	3
PBSC	19
Relapse, *n*	2
Days until relapse: median (range)	500.5 (386–615)
CMV reactivation, *n*	10
Days until CMV reactivation: median (range)	39 (25–102)
ATG: yes/no, *n*	12/10
GvHD prophylaxis, *n*	
Cyclosporin + methothrexate	16
Post‐transplant Cy	2
Tacrolimus + sirolimus	3
Tacrolimus	1
aGVHD, 0/I/II/III	9/3/9/1
Days until aGVHD onset: median (range)	50 (12–162)
cGVHD, none/mild/moderate/severe	8/6/7/1
Days until cGVHD onset: median (range)	195.5 (92–960)
TNC dose (×10^8^/kg), median (range)	8.45 (3.3–20.9)
CD34+ dose (×10^6^/kg), median (range)	62 (7.7–99)
Highest recorded chimerism in the blood, CD3 > 100 days (% of Recipient), median (range)	0.025 (0–12)
Highest recorded chimerism in the bone marrow, CD3 > 100 days (% of Recipient), median (range)	0.3 (0.01–11)

Twenty‐two AML/MDS patients who underwent allogeneic HSCT were included in the study.

aGVHD, acute GvHD; AML, acute myeloid leukaemia; ATG, anti‐thymocyte globulin; BM, bone marrow; cGVHD, chronic GVHD; CMV, cytomegalovirus; Cy, Cyclophosphamide; D, donor; F, Female; GvHD, graft‐versus‐host disease; HLA, human leukocyte antigen; HSCT, haematopoietic stem cell transplantation; M, male; MAC, myeloablative conditioning; MDS, myelodysplastic syndrome; MPN, myeloproliferative neoplasm; MUD, matched unrelated donorl; PBSC, peripheral blood stem cells; R, recipient; RIC, reduced intensity conditioning; TNC, total nucleated cells.

### Sample preparation and flow cytometry (FC)

Peripheral blood mononuclear cells (PBMCs) were isolated by density gradient centrifugation (Ficoll Paque Plus, Cytiva, Chicago, IL, USA) and cryopreserved.^44^ For FC, PBMCs were thawed, washed with PBS, and stained with extracellular antibodies for 20 min at 4°C (Supplementary table 2). After washing, cells were stained with 7‐AAD (BD Biosciences, Franklin Lakes, NJ, USA) for 7 min, resuspended in PBS and acquired on a CytoFLEX using the CytExpert Software v.2.5.0.77 (Beckman Coulter, Brea, CA, USA). FC data were analysed using FlowJo v.10.9 (BD Biosciences).

### Positive selection of γδ+ T‐cells and genomic DNA extraction

γδ+ T‐cells were positively selected from PBMCs using the Anti‐TCRγ/δ MicroBead Kit with MS columns (Miltenyi Biotec, Bergisch Gladbach, Germany) according to manufacturer instructions. Genomic DNA was extracted using the AllPrep DNA/RNA Micro Kit (Qiagen, Hilden, Germany) and stored at −20°C. DNA concentration and purity were determined using NanoDrop2000 (Thermo Fisher Scientific, Waltham, MA, USA). Samples yielding > 0.6 μg DNA were selected for next‐generation sequencing (NGS).

### TRG CDR3 NGS

T‐cell receptor gamma (TRG) CDR3 sequencing was performed as described.^46^ Genomic DNA samples were sequenced by Adaptive Biotechnologies (Seattle, WA). CDR3 amino acid sequences along with corresponding V and J gene segments involved in each rearrangement were annotated according to the IMGT database.^47^, ^48^ The NGS data can be accessed on the Adaptive Biotechnology ImmunoSEQ site (https://doi.org/10.21417/PH2025S). Analysis of TRG repertoire diversity, clonal space homeostasis, segment usage, spectratyping and repertoire overlap was conducted with Immunarch (10.5281/zenodo.3367200) in R and VDJTools (10.1371/journal.pcbi.1004503). Public clones were defined as clones present in more than one donor.

### Graphing and statistical analysis

All data were analysed and visualised using Prism 10 (GraphPad Inc. San Diego, CA, USA). Results were expressed as median ± interquartile range (IQR) or mean ± standard error of the mean (SEM), as appropriate. Two‐sided tests were used. Comparisons between two time points were made using the Wilcoxon test, while comparisons between multiple time points were performed using the Kruskal–Wallis test followed by Dunn's multiple comparisons test. Differences between two cellular populations were assessed using either the Wilcoxon or Mann–Whitney test, with Bonferroni multiple comparisons corrections applied where appropriate. Linear regression and Spearman's correlation analysis were performed where indicated. Significance was set at *P* < 0.05.

## Author contributions

**Paula Hahn:** Data curation; formal analysis; writing – original draft; writing – review and editing; investigation. **Arwen Stikvoort:** Writing – original draft; writing – review and editing; supervision; validation. **Faisal Alagrafi:** Writing – review and editing; writing – original draft; funding acquisition. **Ahmed Gaballa:** Conceptualization; methodology; writing – review and editing. **Martin Solders:** Investigation; data curation; writing – review and editing. **Sofie Vonlanthen:** Investigation; data curation; writing – review and editing. **Johan Karlsson Törlén:** Writing – review and editing; data curation; investigation. **Lucas C M Arruda:** Conceptualization; validation; formal analysis; data curation; writing – original draft. **Thomas Poiret:** Data curation; formal analysis; supervision; writing – original draft; writing – review and editing; validation. **Michael Uhlin:** Conceptualization; resources; funding acquisition; validation; investigation; supervision; writing – review and editing; project administration.

## Conflict of interest

The authors declare no competing interest.

## Supporting information


Supplementary table 1
Supplementary table 2
Supplementary figure 1
Supplementary figure 2
Supplementary figure 3
Supplementary figure 4
Supplementary figure 5
Supplementary figure 6
Supplementary figure 7
Supplementary figure 8
Supplementary figure 9

## Data Availability

The data that support the findings of this study are openly available in immuneACCESS at https://doi.org/10.21417/PH2025S.
