# Supplementary material for: Shared donor–recipient γδ T‐cell phenotypic and repertoire features associate with cytomegalovirus reactivation after allogeneic haematopoietic stem cell transplantation
Source: Clin Transl Immunology. 2025 Dec 17;14(12):e70068. doi: 10.1002/cti2.70068 (PMC12710090; doi:10.1002/cti2.70068)

Supplementary Table S1

Patient #	Flow cytometry					NGS			
	Pre	Donor	1M	3M	12M	Pre	Donor	2M	6M
1						x		x	x
2			x	x	x	x		x	x
3			x	x		x		x	x
4	x		x	x		x		x	x
5			x	x		x		x	x
6			x	x	x	x		x	x
7	x		x	x		x		x	x
8			x	x	x	x	x	x	x
9			x	x	x	x		x	
10			x	x		x		x	
11			x	x		x	x	x	
12	x	x	x	x	x	x		x	
13		x	x	x	x	x	x	x	
14		x	x	x	x				
15	x	x	x	x	x				
16		x	x	x					
17		x	x	x	x				
18	x		x	x	x				
19			x	x	x				
20	x	x	x	x	x				
21	x	x	x	x	x				
22	x		x	x					
**Total**	**8**	**8**	**21**	**21**	**13**	**13**	**3**	**13**	**8**

Supplementary Table S2

Fluorochrome	Marker / Dye	Cat #	Clone	Vendor	Panel
	7-AAD	559925		BD Biosciences	1, 2, 3, 4
Alexa Fluor 700	CD4	557922	RPA-T4	BD Biosciences	1
APC-A700	CD127	A71116	R34.34	Beckman Coulter	2
Alexa Fluor 700	CD86	561124	2331 (FUN-	BD Biosciences	3, 4
APC-Cy7	CD8	557834	SK1	BD Biosciences	1, 2, 4
APC-H7	HLA-DR	561358	G46-6 (L243)	BD Biosciences	3
APC	CD45RO	559865	UCHL1	BD Biosciences	1
APC	NKp44 (CD336)	325110	P44-8	Biolegend	2
APC	NKG2A (CD159a)	130-113-563	REA110	Miltenyi Biotec	3
APC	CCR9 (CD199)	358908	L053E8	Biolegend	4
VioBlue	TCR Vδ2	130-101-157	123R3	Miltenyi Biotec	1, 2, 3
BV421	PD1 (CD279)	564323	MIH4	BD Biosciences	4
BV510	CD3	563109	UCHT1	BD Biosciences	1, 2, 3, 4
BV650	CCR2 (CD192)	747849	LS132.1D9	BD Biosciences	1
BV650	CX3CR1	341625	2A9-1	Biolegend	2
BV650	NKG2D (CD314)	563408	1D11	BD Biosciences	3
BV650	LAG3 (CD223)	369316	11C3C65	Biolegend	4
BV785	DNAM-1 (CD226)	338322	11A8	Biolegend	1
BV786	CD69	563834	FN50	BD Biosciences	2, 4
BV785	CXCR3 (CD183)	353737	G025H7	Biolegend	3
FITC	TCR Vδ1	TCR2730	TS8.2	Invitrogen	1,3,4
FITC	TCR Vγ9	331306	B3	Biolegend	2
PE	TCR γδ	130-113-512	REA591	Miltenyi Biotec	1, 2, 3, 4
PE Vio770	CD27	130-113-631	M-T271	Miltenyi Biotec	1
PE Vio770	CD39	130-110-790	REA739	Miltenyi Biotec	2
PE Vio770	NKG2C (CD159c)	130-120-589	REA205	Miltenyi Biotec	3
PE Cy7	CD158b/j	312610	DX27	Biolegend	4
PE-CF594	CCR7 (CD197)	562381	150503	BD Biosciences	1
PE-CF594	CD28	222.562296	CD28.2	BD Biosciences	2
PE-CF594	CCR6 (CD196)	564816	11A9	BD Biosciences	3
PE-CF594	TIM3 (CD366)	565561	7D3	BD Biosciences	4

Supplementary Figure S1

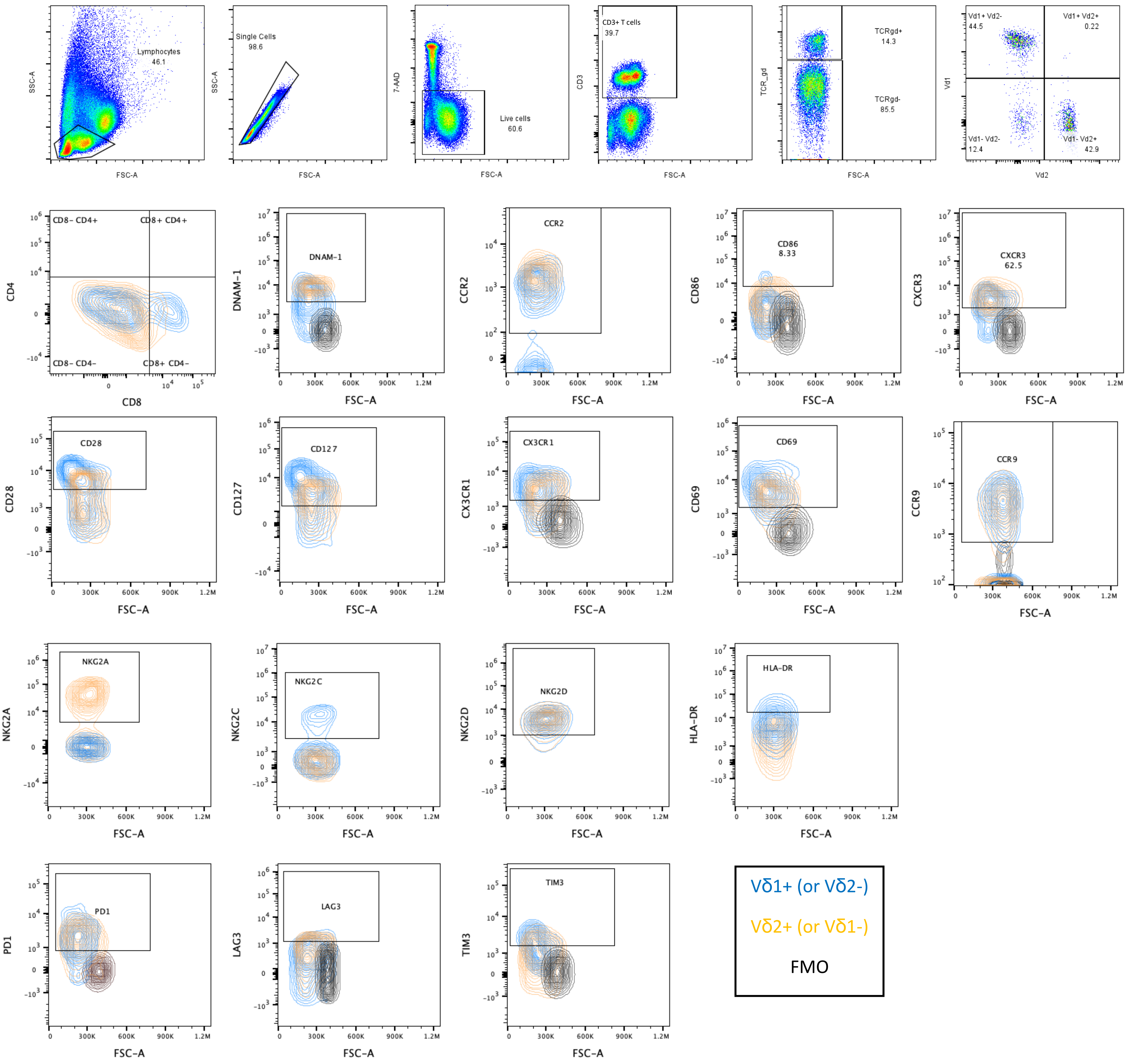

Supplementary Figure S2

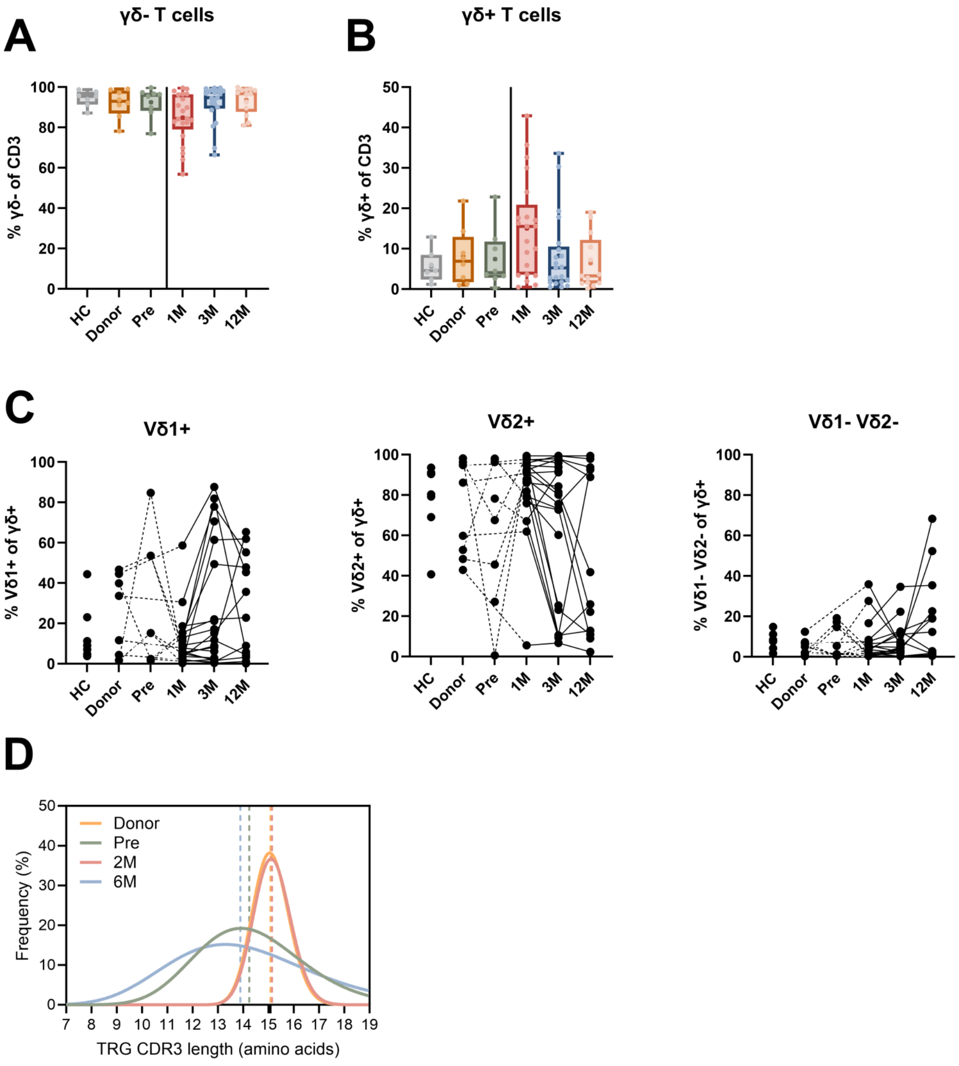

Supplementary Figure S3

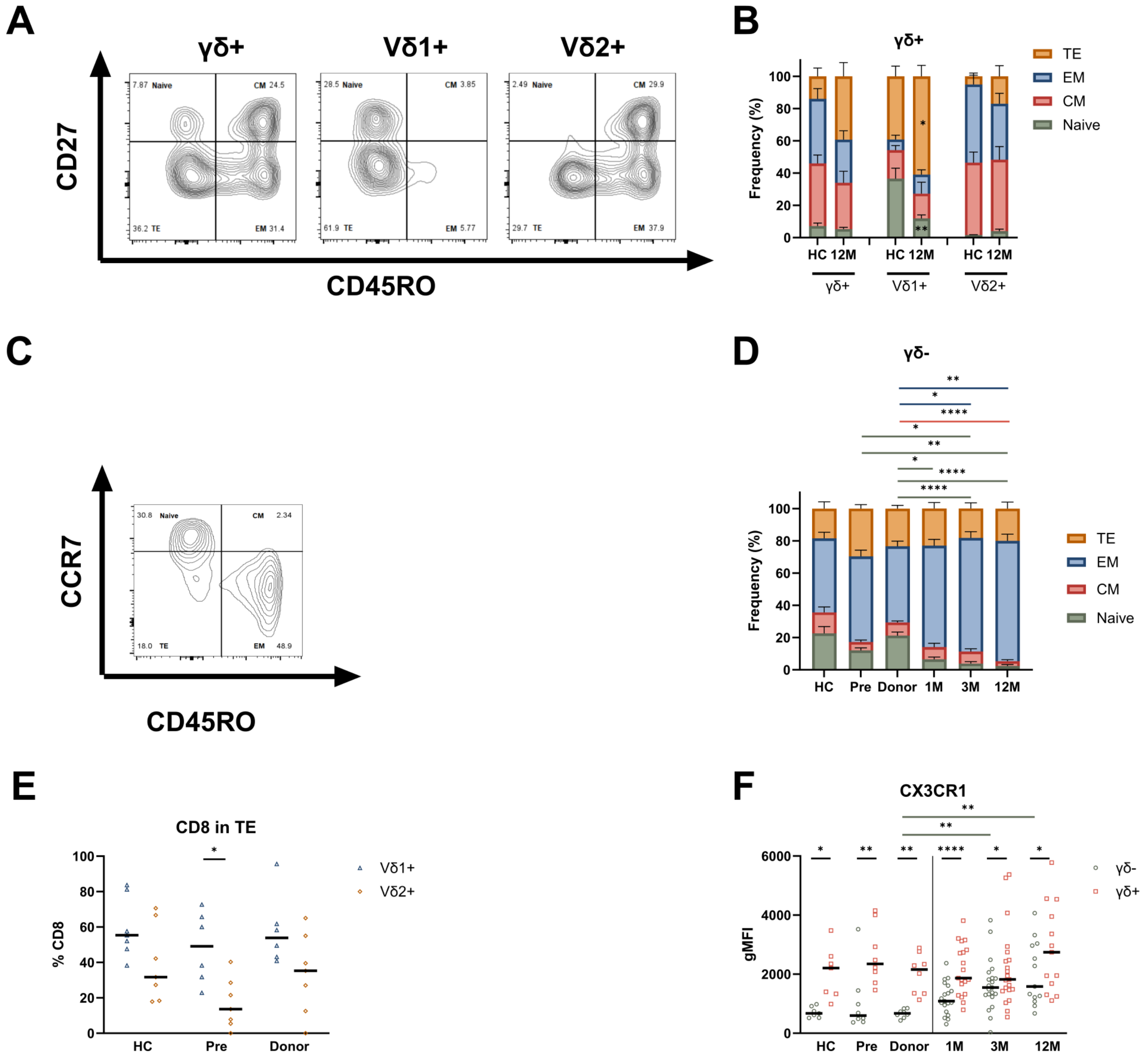

Supplementary Figure S4

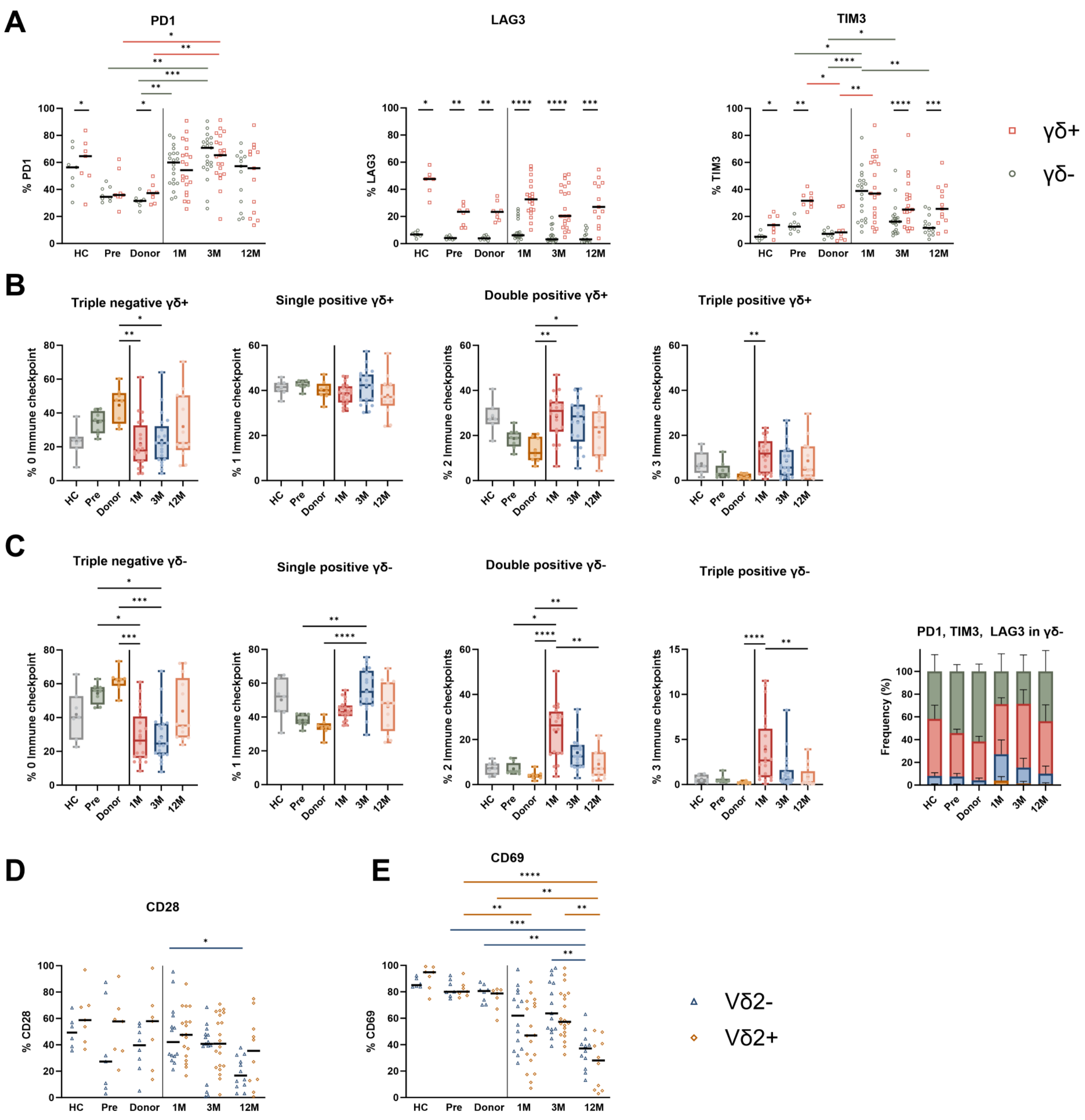

Supplementary Figure S5

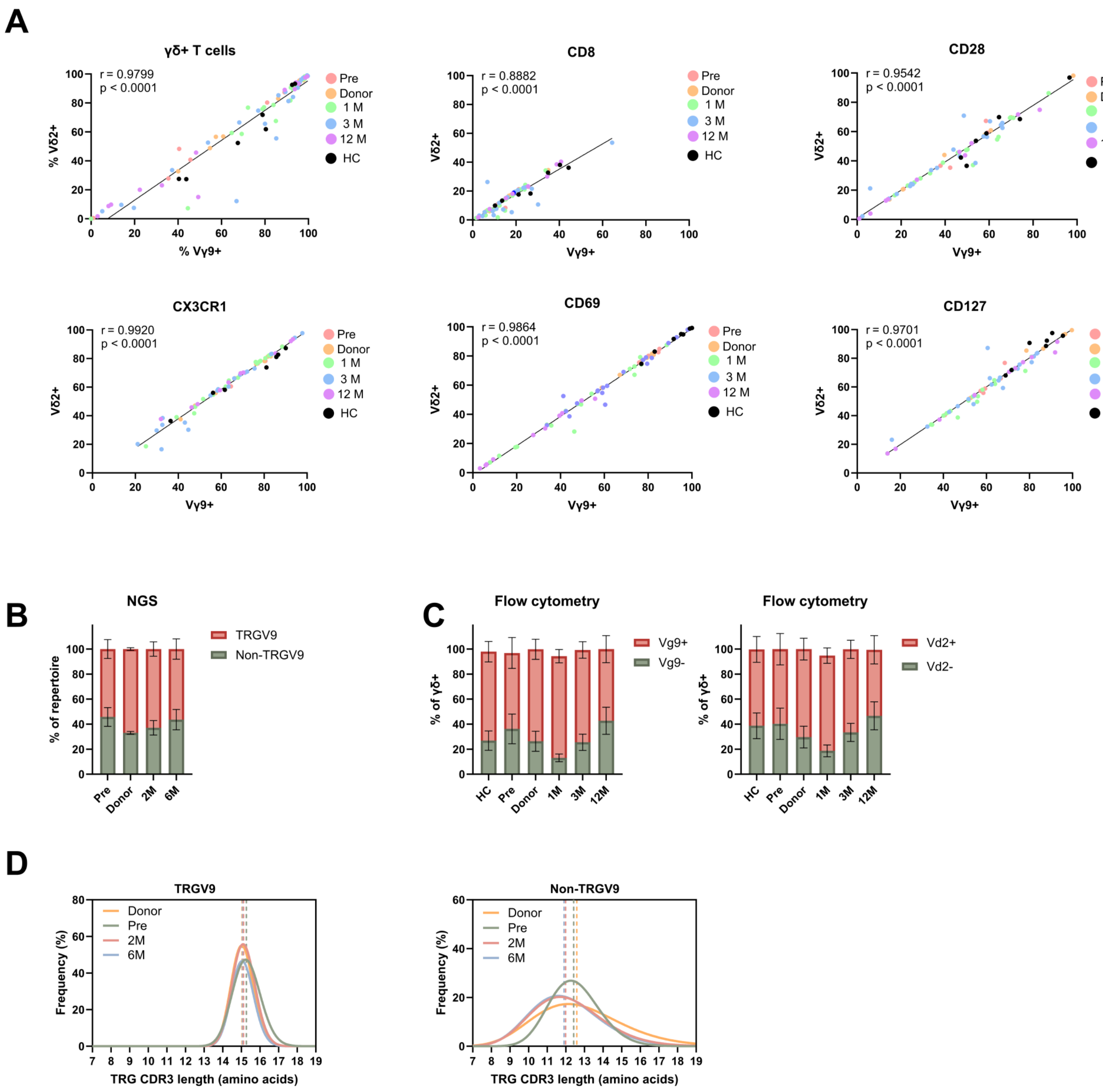

Supplementary Figure S6

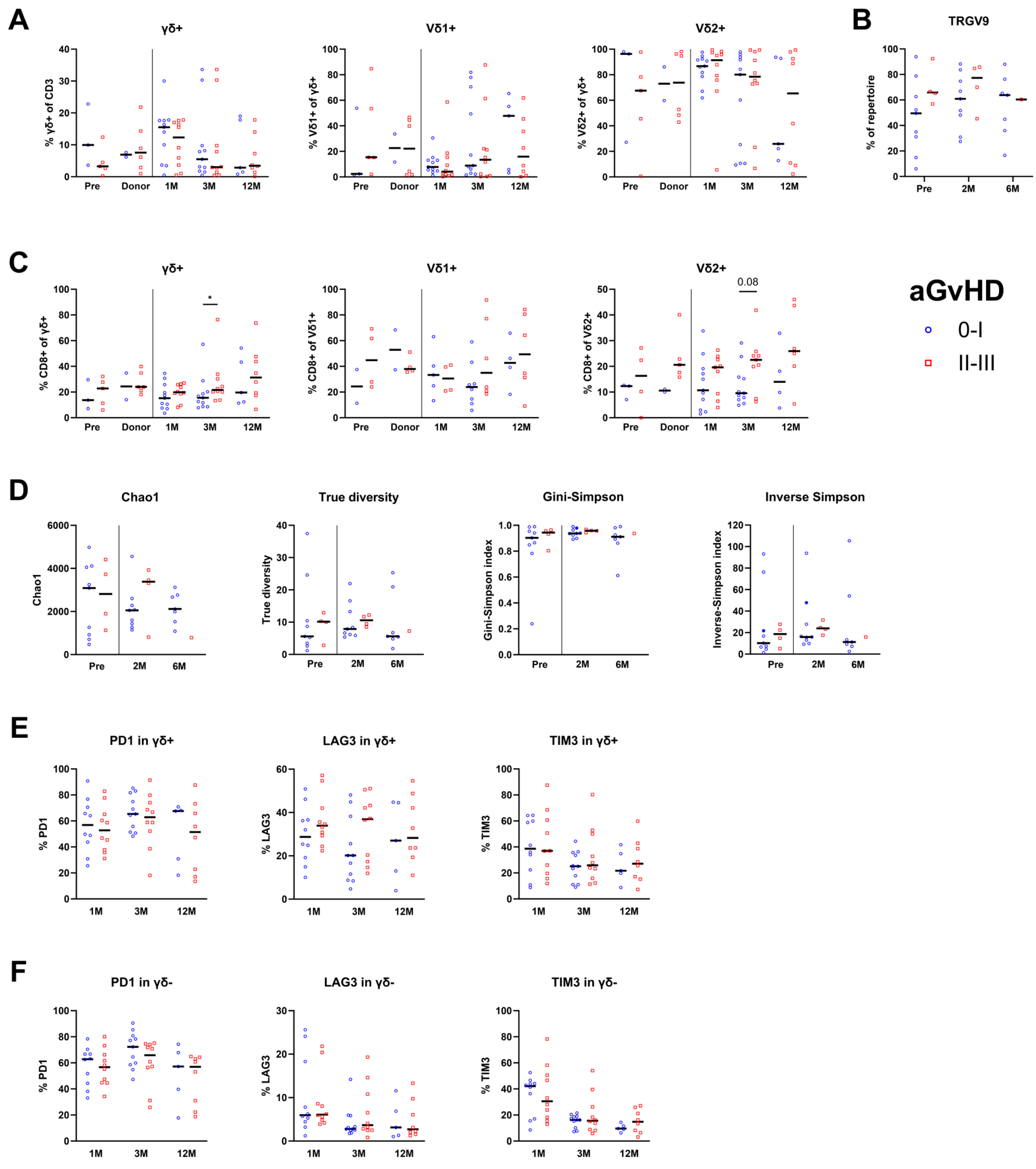

Supplementary Figure S7

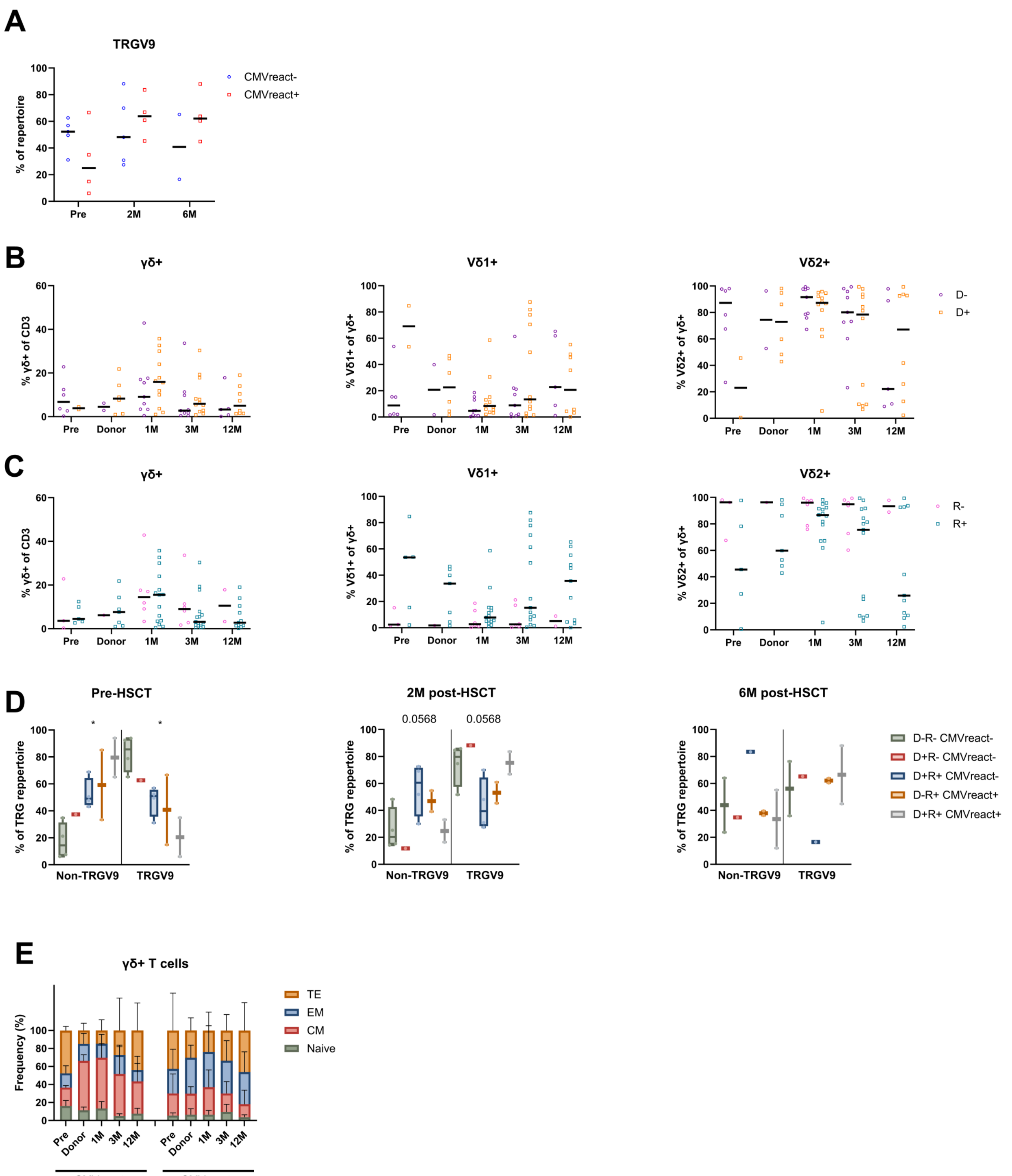

Supplementary Figure S8

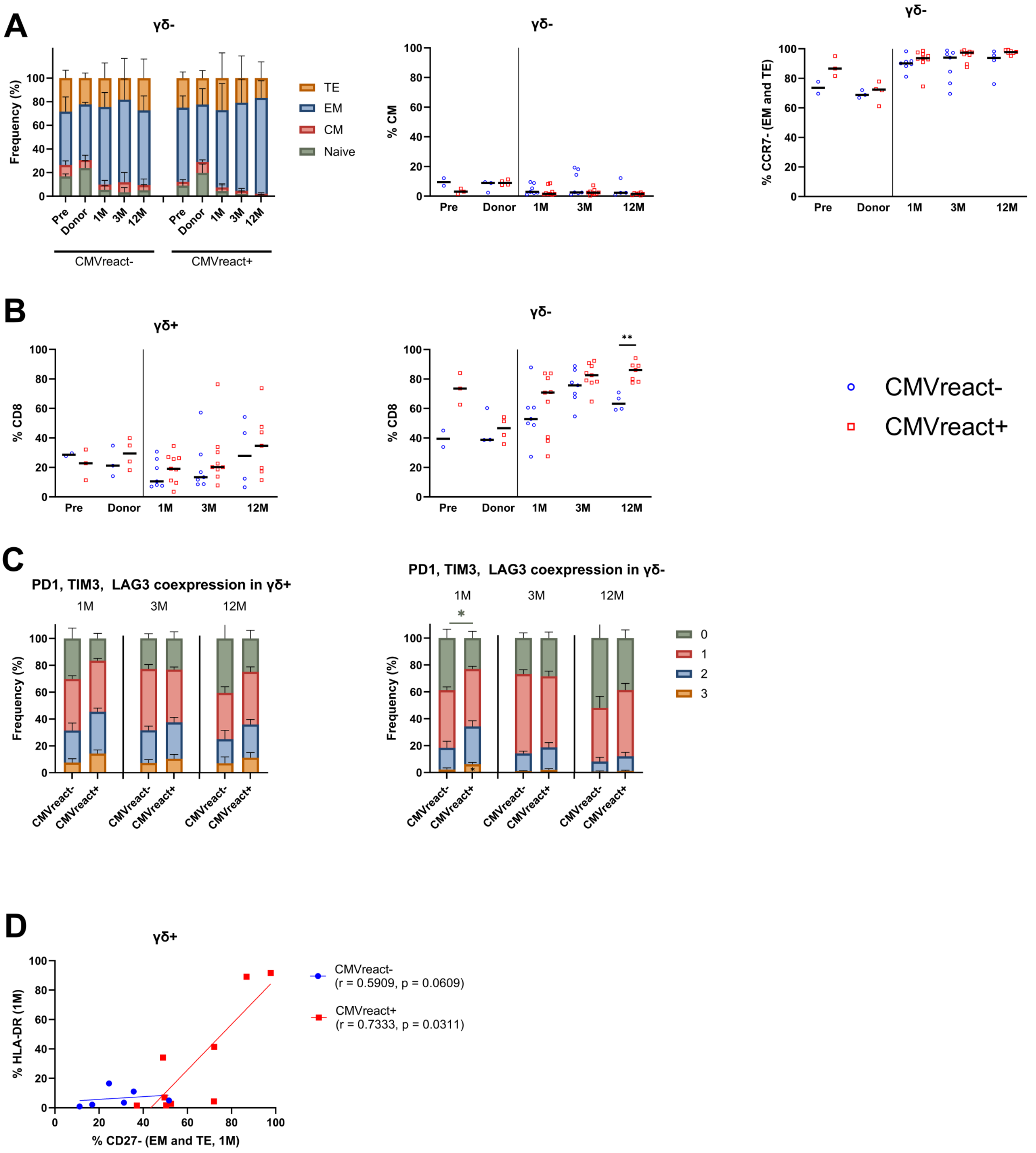

Supplementary Figure S9

All chains

CMVreact+

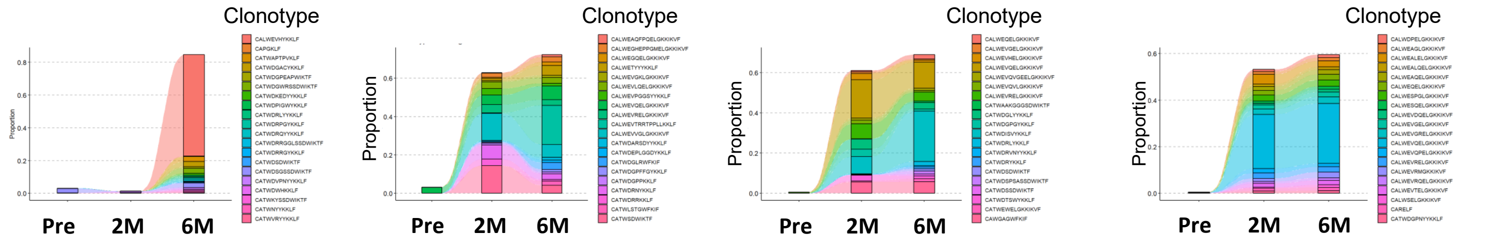

CMVreact-

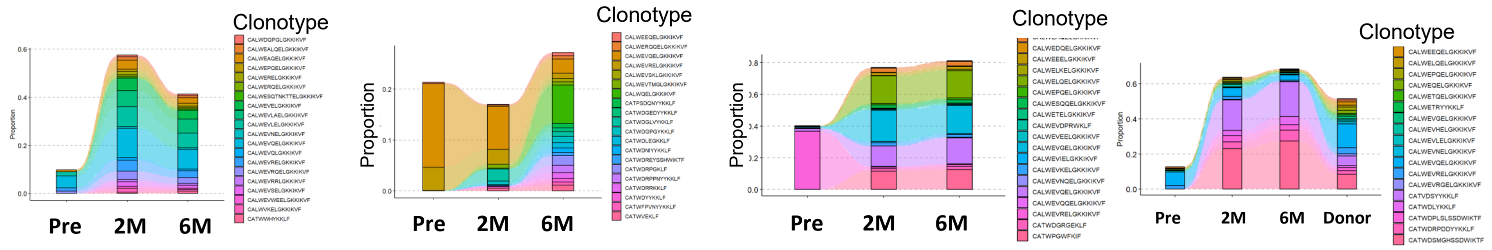

TRGV9

CMVreact+

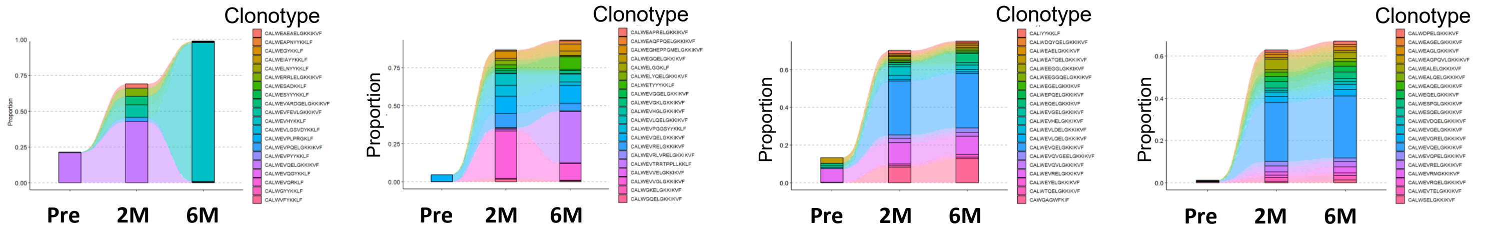

CMVreact-

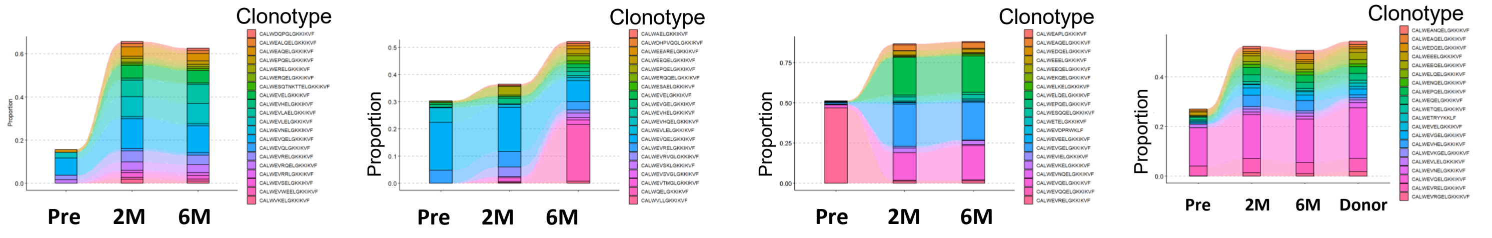

Non-TRGV9

CMVreact+

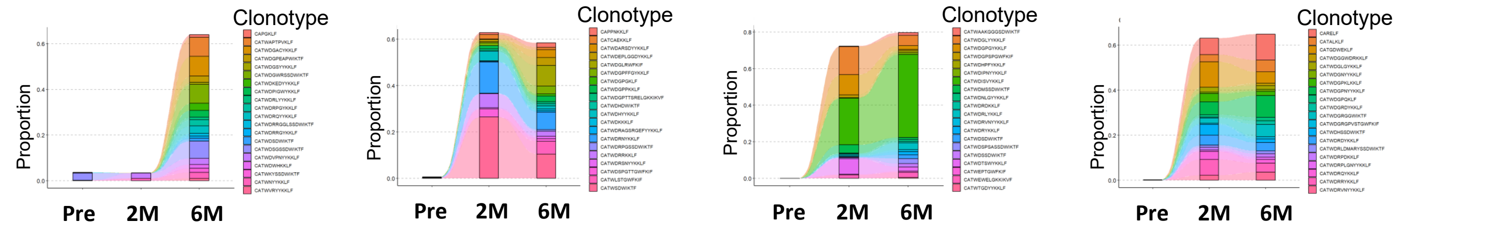

CMVreact-

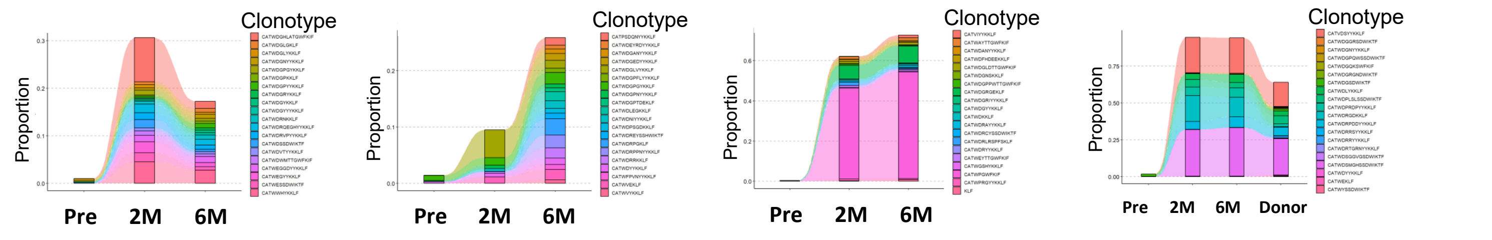

Supplement: Supplementary file 1 — Supplementary table 1 Supplementary table 2 Supplementary figure 1 Supplementary figure 2 Supplementary figure 3 Supplementary figure 4 Supplementary figure 5 Supplementary figure 6 Supplementary figure 7 Supplementary figure 8 Supplementary figure 9 [file CTI2-14-e70068-s001.zip › cti270068-sup-0001-Supinfo.pdf]
