# Supplementary material for: Shared donor–recipient γδ T‐cell phenotypic and repertoire features associate with cytomegalovirus reactivation after allogeneic haematopoietic stem cell transplantation
Source: Clin Transl Immunology. 2025 Dec 17;14(12):e70068. doi: 10.1002/cti2.70068 (PMC12710090; doi:10.1002/cti2.70068)
Supplement: Supplementary file 1 — Supplementary table 1 Supplementary table 2 Supplementary figure 1 Supplementary figure 2 Supplementary figure 3 Supplementary figure 4 Supplementary figure 5 Supplementary figure 6 Supplementary figure 7 Supplementary figure 8 Supplementary figure 9 [file CTI2-14-e70068-s001.zip › 3_251201_Supplementary materials_Legends_All_edits_Clean.docx]

**Supplementary materials**

**Supplementary Table S1. Patient samples used for FC and NGS**.

**Supplementary Table S2. List of FC antibodies and panels.**

**Supplementary Figure S1. Gating strategies to identify γδ T-cells and subsets.** Representative flow cytometry plots showing the gating strategies to identify γδ+ and γδ- T-cells from CD3+ T cells, and the Vδ1+ and Vδ2+ subsets from γδ+ T cells. Further gating to characterize the phenotype was performed in both γδ+ T cell subsets, with the help of fluorescence minus one (FMO) controls.

**Supplementary Figure S2. T-cell subsets early post-HSCT.** Frequency of γδ− (**A**) and γδ+ (**B**) T-cells from CD3+ cells in controls (HC, Donor, Pre-HSCT) and at 1, 3 and 12 months post-HSCT. Range is shown. **(C)** Individual patient-donor pairs frequency of Vδ1+, Vδ2+ and Vδ1- Vδ2- γδ T-cells. **(D)** CDR3 length distribution (in amino acids), displayed as the relative proportion of CDR3 sequences for each length weighed by the number of clones per clonotype. The curves represent nonlinear curve fitting (Gauss function) applied to the frequency of TRG lengths in each group. Vertical lines denote the mean CDR3 length for each group. HC: healthy controls, Pre: pre-HSCT, DN: Vδ1− Vδ2−. 3 < N < 21.

**Supplementary Figure S3. Differentiation of γδ+ and γδ− T-cells post-HSCT. (A)** Representative flow cytometry plots of differentiation subsets of γδ+ T-cells defined by CD27 and CD45RO. **(B)** Comparison of differentiation states between HC and 12M post-HSCT. **(C)** Representative FC plots of differentiation states of γδ− T-cells defined by CCR7 and CD45RO. **(D)** Differentiation states of γδ− T-cells. **(E)** Frequency of CD8 in TE cell subsets in controls (HC, Pre, and Donor). **(F)** CX3CR1 expression (gMFI) in γδ− and γδ+ T-cells. **(B)** Mann-Whitney test was used to compare between HC and 12M in each population and cell subset. **(D)** Kruskal-Wallis test with Dunn’s multiple comparisons to compare timepoints within populations, excluding HC. **(E)** Mann-Whitney test to compare Vδ1+ and Vδ2+ cell populations. **(F)** Wilcoxon test to compare γδ+ and γδ− cell populations, Kruskal-Wallis test with Dunn’s multiple comparisons to compare timepoints, excluding HC. HC: healthy controls, Pre: pre-HSCT, CM: central memory phenotype, EM: effector memory, TE: terminal effector, Pre: pre-HSCT, gMFI: geometric mean fluorescence intensity. 6 < N < 21.

**Supplementary Figure S4. Inhibitory and γδ and γδ− T-cell specific phenotype post-HSCT. (A)** Expression of PD1, LAG3 and TIM3 in γδ+ and γδ− T-cells. Number of co-expression of immune checkpoint markers (PD1, LAG3, and TIM3) in γδ+ **(B)** and in γδ− **(C)** T-cells. Expression of CD28 **(D)** and CD69 **(E)** in Vδ2- and Vδ2+ cell subsets. **(A)** Wilcoxon test to compare subsets and Kruskal-Wallis test with Dunn’s multiple comparisons to compare timepoints (excluding HC). **(B-C)** Kruskall-Wallis test with Dunn’s multiple comparisons test, excluding HC. **(D-E)**. Mann-Whitney test to compare cell subsets and Kruskal-Wallis test with Dunn’s multiple comparisons to compare timepoints (excluding HC). Medians are represented. **P* < 0.05, ***P* < 0.01, ****P* < 0.001, *****P* < 0.0001. HC: healthy control, Pre: pre-HSCT. 6 < N < 21.

**Supplementary Figure S5. Similar profile of Vδ2+ and Vγ9+ cells. (A)** Spearman correlation between Vδ2+ and Vγ9+ frequency (first panel) and frequency of CD8, CD28, CX3CR1, CD69 and CD127 on Vδ2+ and Vγ9+ cell subsets assessed by flow cytometry. Linear regression line is shown. **(B)** Distribution of TRGV9 and non-TRGV9 clones across timepoints post-HSCT assessed by next-generation sequencing (NGS). **(C)** Distribution of Vγ9+ and Vγ9− cells and of Vδ2+ and Vδ2− cells across timepoints assessed by flow cytometry. **(D)** CDR3 length distribution (in amino acids), displayed as the relative proportion of CDR3 sequences for each length weighed by the number of clones per clonotype of the TRGV9 repertoire (left) and the non-TRGV9 repertoire (right). The curves represent nonlinear curve fitting (Gauss function) applied to the frequency of TRG lengths in each group. Vertical lines denote the mean CDR3 length for each group. HC: healthy controls, Pre: pre-HSCT. 8 < N < 21.

**Supplementary Figure S6. Acute graft-versus-host disease is not associated with γδ T-cell phenotype diversity, or inhibitory phenotype. (A)** Frequency of γδ+, Vδ1+ and Vδ2+ T-cells by aGVHD grading. **(B)** Frequency of TRGV9 chains among the repertoire by aGvHD grading. **(C)** Frequency of CD8-expressing γδ+ T-cells, Vδ1+ cells and Vδ2+ cells by aGVHD. **(D)** Chao-1 index, true diversity index, Gini-Simpson index and inverse Simpson index of γδ+ TCR by aGVHD. Frequency of PD1-, LAG3- and TIM3-expressing cells in γδ+ (**E**) and γδ− (**F**) T-cells in patients by aGVHD grading. (**A-F**) Mann Whitney test (grade 0-I vs. grade II-III aGVHD within a timepoint). Medians are represented. **P* < 0.05. aGVHD: acute graft-versus-host disease, Pre: pre-HSCT. 1 < N < 11.

**Supplementary Figure S7. Comparison of γδ T-cells by CMV serostatus and reactivation. (A)** Comparison of TRGV9 frequency of repertoire in patients by CMV reactivation (excluding D-R-). **(B)** Comparison of frequency of γδ+ T-cells (left), Vδ1+ T-cells (middle) and Vδ2+ T-cells (right) between patients receiving grafts from CMV-seronegative (D-) and seropositive (D+) donors. **(C)** Comparison of frequency of γδ+ T-cells (left), Vδ1+ T-cells (middle) and Vδ2+ T-cells (right) between CMV-seronegative (R-) and seropositive (R+) graft recipients. **(D)** Comparison of non-TRGV9 and TRGV9 frequency of repertoire in patients by CMV-serostatus and CMV reactivation pre-HSCT and at 2 and 6 months post-HSCT. (**E**) Differentiation states of γδ+ T-cells by CMV reactivation groups (excluding D-R-). **(A-C)** Mann-Whitney test. **(D)** Kruskal-Wallis test for Pre and 2M. Medians are represented. **P* < 0.05, ***P* < 0.01, ****P* < 0.001. CM: central memory; EM: effector memory; TE: terminal effector, Pre: pre-HSCT. 3 < N < 12.

**Supplementary Figure S8. γδ+ and γδ− T-cell phenotype and CMV reactivation. (A)** Differentiation states of γδ− T-cells by CMV reactivation (excluding D-R-). Mean with SEM (stacked bar chart) and median (scatter plot). **(B)** Frequency of CD8 expressing γδ+ and γδ− T-cells by CMV reactivation (excluding D-R-). Median is shown. **(C)** Comparison of number of immune checkpoint molecules (PD1, TIM3 and LAG-3) co-expressed post-HSCT by CMV-reactivation in γδ+ T-cell and γδ− T-cells (excluding D-R-). Mean with SEM. **(D)** Spearman correlation of frequency of HLA-DR and frequency of CD27− (EM and TE subsets) at 1M post-HSCT in γδ+ T-cells by CMV reactivation (excluding R-D-). Simple linear regression line is shown. Medians are represented. **(A-B)** Mann-Whitney test to compare patient groups. **P* < 0.05, ***P* < 0.01. Pre: pre-HSCT; CM: central memory; EM: effector memory; TE: terminal effector. 3 < N < 12.

**Supplementary Figure S9. Clonotype tracking of the top 20 TRGV9 or non-TRGV9 clonotypes present at 6M-post HSCT in patients with or without CMV reactivation.**
